# Active bio-packaging with PHBHHx-ZnO bionanocomposites: advancing food safety and shelf-life

**DOI:** 10.3389/fnut.2026.1789448

**Published:** 2026-03-18

**Authors:** Lutfun Nahar, Satyajit D. Sarker

**Affiliations:** 1 Laboratory of Growth Regulators, Palacký University and Institute of Experimental Botany, The Czech Academy of Sciences, Olomouc, Czechia; 2 Centre for Natural Products Discovery, School of Pharmacy and Biomolecular Sciences, Liverpool John Moores University, Liverpool, United Kingdom

**Keywords:** active packaging, bionanocomposites, food shelf-life, PHBHHx, sustainability

## Abstract

Food loss and waste remain major global challenges. Perishables like fish, fruits, and vegetables show the highest post-harvest losses. Conventional petroleum-based packaging offers limited preservation while adding long-term pollution, and recent assessments indicate that more than one billion tons of food were wasted in 2022, reinforcing the need for improved preservation strategies. Poly(3-hydroxybutyrate-co-3-hydroxyhexanoate) (PHBHHx) films reinforced with ZnO nanoparticles provide flexibility, biodegradability, and reliable processing. ZnO increases mechanical stiffness and thermal stability, strengthens oxygen-barrier and antimicrobial functions, and delivers strong UV shielding. These combined properties define the active-preservation behavior of PHBHHx-ZnO films and support their relevance for chilled food systems. ZnO acts as a nucleating agent, with PHBHHx-ZnO films typically showing crystallinity in the 53–56% range without loss of flexibility. Studies with ZnO-enabled active films extend refrigerated shelf life. Storage periods of 6–8 days rise to 12–16 days in seafood models, with similar improvements for meat and dairy products. These gains arise from nanoparticle-polymer interactions that increase crystallinity, restrict polymer mobility, and stabilize film microstructures. PHBHHx-ZnO bionanocomposites offer a promising route toward safe, active, and sustainable packaging systems. This review outlines further directions, including migration-compliant ZnO placement, lower-impact ZnO preparation routes, and multifunctional designs aligned with more circular-economy goals.

## 1 Introduction

Global pressures from plastic pollution and food waste continue to shape packaging priorities, with persistent plastics and high food-loss rates driving interest in safer, renewable materials (1). International assessments highlight the scale of the challenge, with food-loss indicators and waste-index reports underscoring the need for packaging that reduces spoilage and supports sustainable supply chains (2, 3, 57). Updated global estimates reported more than one billion tons of food wasted in 2022, equivalent to 79 kg of household waste per person per year and 8–10% of global greenhouse-gas emissions, which highlights the scale of the preservation challenge that active bio-packaging aims to address. Recent work shows how biopolymers and natural compounds contribute to these transitions through biodegradability, functional performance, and alignment with circular-economy goals (1, 4, 5). Early demonstrations of antimicrobial and UV-protective behavior in biopolymer films, including PHBV-tannin, PLA-ZnO, and cellulose-ZnO systems, reinforced the relevance of active biodegradable packaging for reducing spoilage in perishable foods (6). This context frames the need for clearer evaluation of biodegradable matrices that combine structural performance with active functionality.

Biodegradable polymers now attract strong attention as alternatives to petroleum-based plastics due to rising environmental concerns, regulatory pressure, and the need for sustainable packaging solutions. Their biodegradability, favorable toxicological profiles, and compatibility with food-contact applications support broader adoption across food systems (7, 8). Reviews on biobased and biodegradable plastics further highlight opportunities for reducing environmental burdens while maintaining functional performance in food-packaging settings (9, 10). Evidence from real food systems shows that biodegradable matrices reinforced with functional additives can reduce microbial loads and slow oxidative deterioration during chilled storage (11, 12). These findings strengthen the case for active biodegradable films that integrate antimicrobial and stabilizing mechanisms.

Polyhydroxyalkanoates (PHAs) represent a major class of microbially derived polyesters with strong biodegradability and stable performance in food-contact settings. Their microbial origin, favorable safety profile, and ability to degrade under composting, soil, and marine conditions strengthen their relevance for sustainable packaging (7, 13). Advances in PHA production from renewable feedstocks, including volatile fatty acids and waste-derived substrates, further support their integration into circular-economy strategies (14, 15). Studies on PHA-based films containing antimicrobial agents demonstrate practical benefits for reducing spoilage in fish, meat and seafood systems (16, 17). These attributes position PHAs as adaptable platforms for active-packaging applications.

PHBHHx occupies a distinctive position within the PHA family due to the presence of 3-hydroxyhexanoate units, which reduce crystallinity, increase flexibility and support broader processing windows compared with PHB and PHBV. These characteristics enable improved mechanical resilience and stable performance in flexible and semirigid packaging formats (18, 19). Studies on PHBH and related copolymers further demonstrate enhanced toughness and compatibility with nanofillers, reinforcing the suitability of PHBHHx for advanced packaging applications (20, 21). Processing advances show that PHBHHx supports multilayer structures and uniform nanoparticle dispersion, strengthening its role in active packaging (19, 22). This combination of flexibility, compatibility and processing stability makes PHBHHx a strong candidate for next-generation bionanocomposites.

Zinc oxide (ZnO) has emerged as a multifunctional nanofiller that strengthens biodegradable matrices through antimicrobial activity, UV absorption, photocatalytic behavior and nucleation effects (6, 23). These functions arise from ROS generation, Zn^2+^ release, membrane disruption, wide-bandgap UV absorption and particle-size-dependent optical behavior. These properties address the main deterioration pathways in fish, meat, dairy and fresh-produce systems, where microbial growth, oxidative reactions and light exposure drive rapid quality loss (4, 24, 25).

ZnO also increases crystallinity, improves thermal stability and enhances mechanical behavior in PHA, PLA, starch, cellulose and chitosan matrices (26–28). Crystallinity increases of 3–12% and decomposition-onset shifts of 10–25 °C have been reported across PHAs and PLA, confirming consistent nucleation and stabilization effects. Recent reviews on ZnO synthesis and food-packaging applications further highlight its versatility and safety profile (29, 30). Real-food studies confirm that ZnO-reinforced films can extend shelf life and reduce spoilage in beverages, seafood, and meat products (11, 12, 16). Its multifunctionality provides synergistic advantages when combined with biodegradable matrices.

PHBHHx-ZnO systems show strong potential for active-packaging applications that require antimicrobial activity, UV protection, and oxidative-stability control. Studies in fish, meat, dairy, and bakery products report significant reductions in microbial loads, slower lipid oxidation, and improved sensory quality when ZnO-reinforced biodegradable films are used during refrigerated storage (11, 16, 17). These improvements appear at low nanoparticle concentrations, supporting safe integration into food-contact materials.

PHBHHx provides a flexible and biodegradable matrix that supports uniform nanoparticle dispersion and consistent functional performance across a wide range of food categories. Comparative studies across PLA, PHBV, starch, cellulose, chitosan, and PCL confirm the balanced performance profile of PHBHHx-ZnO systems (31–34). Evidence from PHBHHx-ZnO films shows stable crystallinity, strong UV and oxygen-barrier gains and confirmed antibacterial activity, reinforcing their suitability for chilled food storage (22). Oxygen-transmission reductions of 15–40% and UV-transmittance reductions of 60–95% have been reported at low ZnO loadings, supporting their practical relevance. These combined advantages support their development as practical active-packaging materials.

Current trends emphasize greener ZnO-preparation routes, surface-engineered nanoparticles, hybrid structures, multilayer formats, and smart-packaging systems (23, 29, 35). These developments align with global sustainability priorities and support integration into circular-economy strategies. Key challenges remain in nanoparticle dispersion, migration control, environmental fate, and regulatory approval, but advances in surface engineering, controlled-release systems, and multilayer structures provide clear pathways for future development. Migration-safety considerations, including ionic Zn^2+^ limits and nanoparticle-confinement strategies, increasingly shape the design of active biodegradable packaging (23, 29, 35). This review evaluates structural, functional, and ecological evidence to define the performance landscape of PHBHHx-ZnO systems and to clarify their role in active, sustainable food-packaging applications.

## 2 ZnO nanoparticles: properties, mechanisms, and relevance for food-packaging systems

### 2.1 Antimicrobial mechanisms

ZnO nanoparticles show broad-spectrum antimicrobial behavior through several coordinated routes. Reactive oxygen species form at the particle surface and injure membranes, proteins, and nucleic acids, while Zn^2+^ release perturbs enzyme activity and ion transport. Direct particle-cell contact further intensifies local membrane damage (30, 36, 37). Trials with biodegradable films in seafood and meat consistently report lower microbial loads during chilled storage at low ZnO loadings, confirming active performance in food-contact matrices (11, 16, 25).

**Figure 1:**
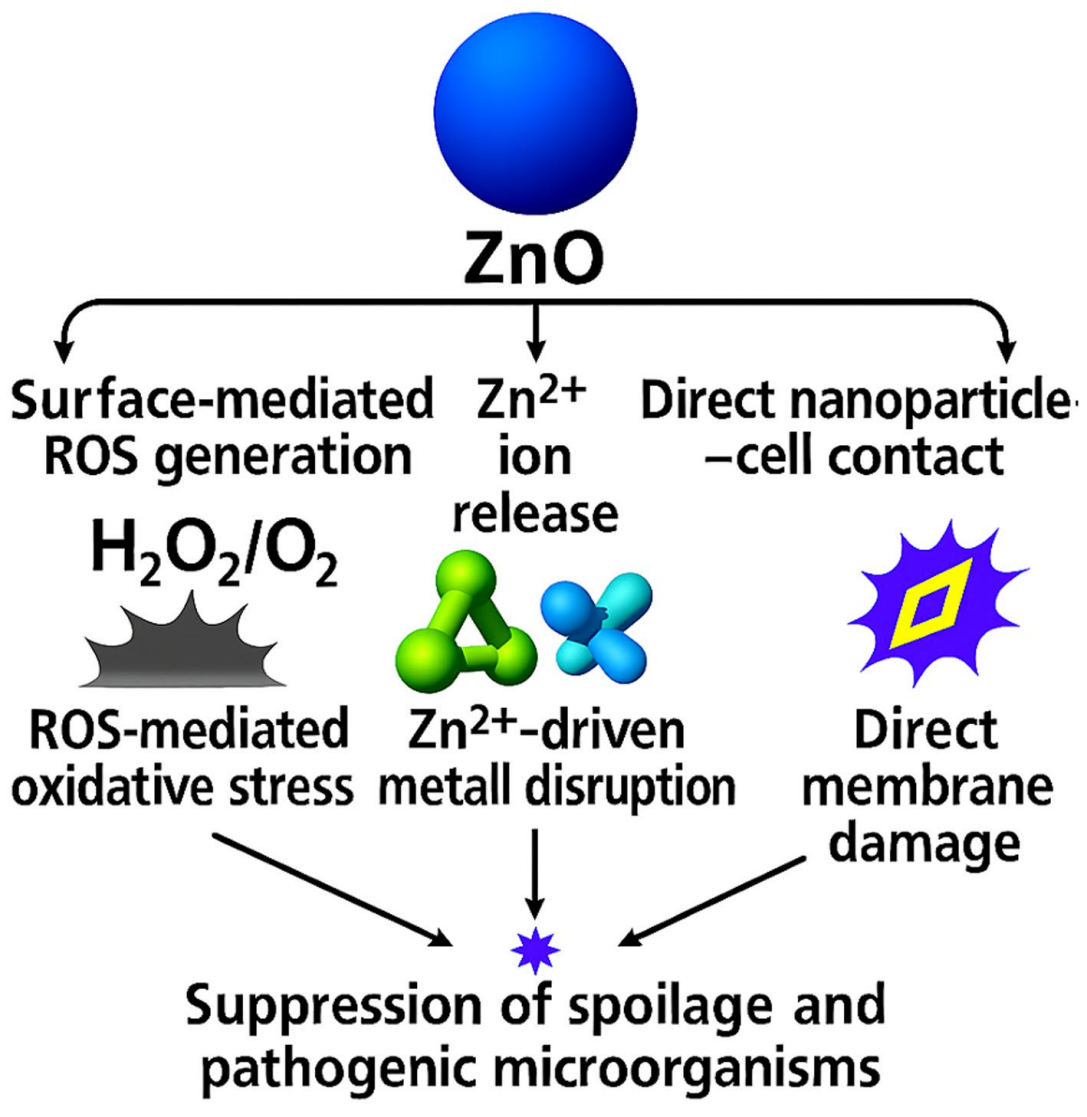
Antimicrobial mechanisms of ZnO nanoparticles in food-packaging systems.

Reductions in total viable counts and common spoilage organisms appear across fish and meat systems under refrigerated conditions, demonstrating stable antimicrobial action in real food environments (11, 16, 25). These effects arise from ROS-mediated oxidative stress, Zn^2+^-driven metabolic disruption, and membrane destabilization, which together suppress microbial proliferation on food surfaces during storage. ZnO nanoparticles exert antimicrobial activity through three coordinated pathways: surface-mediated ROS generation, Zn^2+^ release that disrupts metabolic and enzymatic processes, and direct nanoparticle-cell contact that damages membranes. These mechanisms collectively suppress spoilage and pathogenic microorganisms in chilled fish and meat systems, supporting active preservation at low ZnO loadings (11, 16, 25). Figure 1 illustrates the antimicrobial pathways of ZnO nanoparticles in food-packaging systems.

### 2.2 UV-protective behavior

Wide-bandgap optics define ZnO. Strong absorption in UVA and UVB delivers active UV shielding that reduces photo-oxidation, pigment loss, and light-driven nutrient degradation in dairy, fish, and produce systems (23, 38). Coatings and thin films on PHBHHx, PLA, and PET show marked drops in UV transmittance without sacrificing transparency at modest loadings (23, 38). UV-blocking behavior remains stable across thin-film formats when ZnO dispersion is uniform, supporting active optical protection during storage (23, 38).

**Figure 2:**
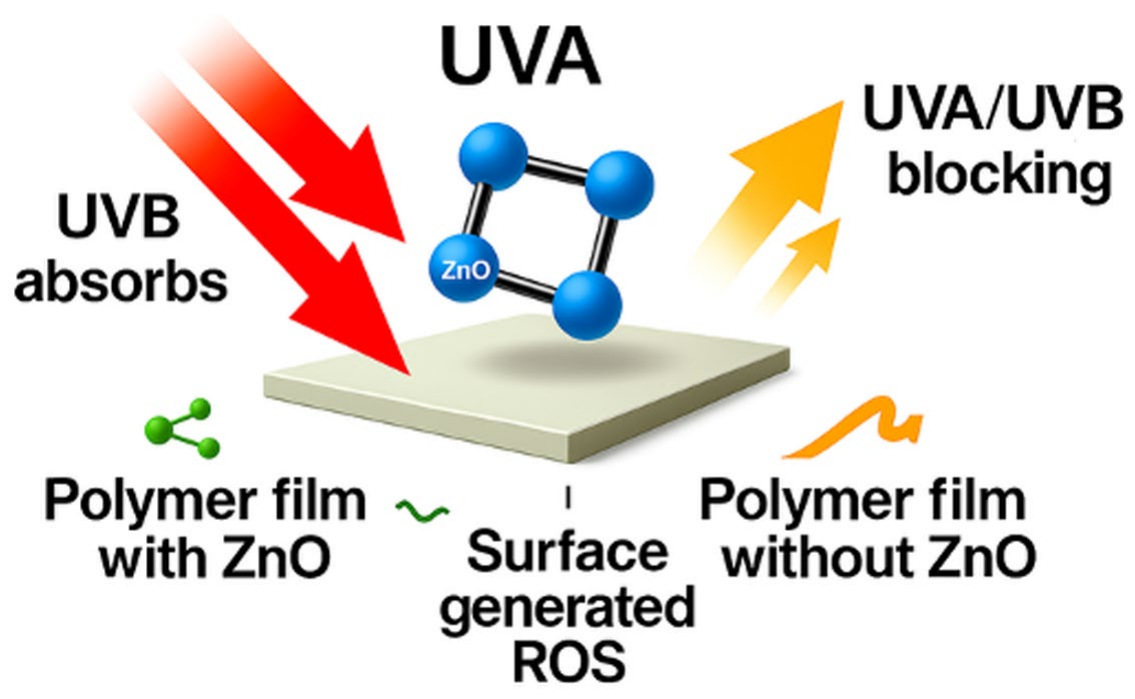
Overview of ZnO’s UV-shielding behavior across common food-packaging matrices.

Particle size and morphology influence absorption efficiency, with smaller particles enhancing UVA/UVB attenuation while maintaining clarity. ZnO’s wide bandgap supports strong UVA and UVB absorption that reduces photo-oxidation, pigment loss, and nutrient degradation in dairy, fish, and produce systems. Uniform dispersion in PHBHHx, PLA, and PET films maintains transparency while delivering effective UV blocking at modest loadings (23, 38). Figure 2 presents ZnO’s UV-shielding behavior across common food-packaging matrices.

### 2.3 Nucleation and crystallinity effects

Nucleation remains a central materials function of ZnO in biopolymer matrices. Particles present as dispersed domains promote ordered chain growth, increase crystallinity, refine spherulite structure, and stiffen films, effects that typically lower oxygen permeability, stabilize thermal behavior, and contribute to active-barrier enhancement in food-packaging applications (26, 28). Crystallinity gains appear at low particle fractions, aligning with nucleation trends in PLA, PHBV, cellulose, and PVA matrices (27, 33, 39).

**Figure 3:**
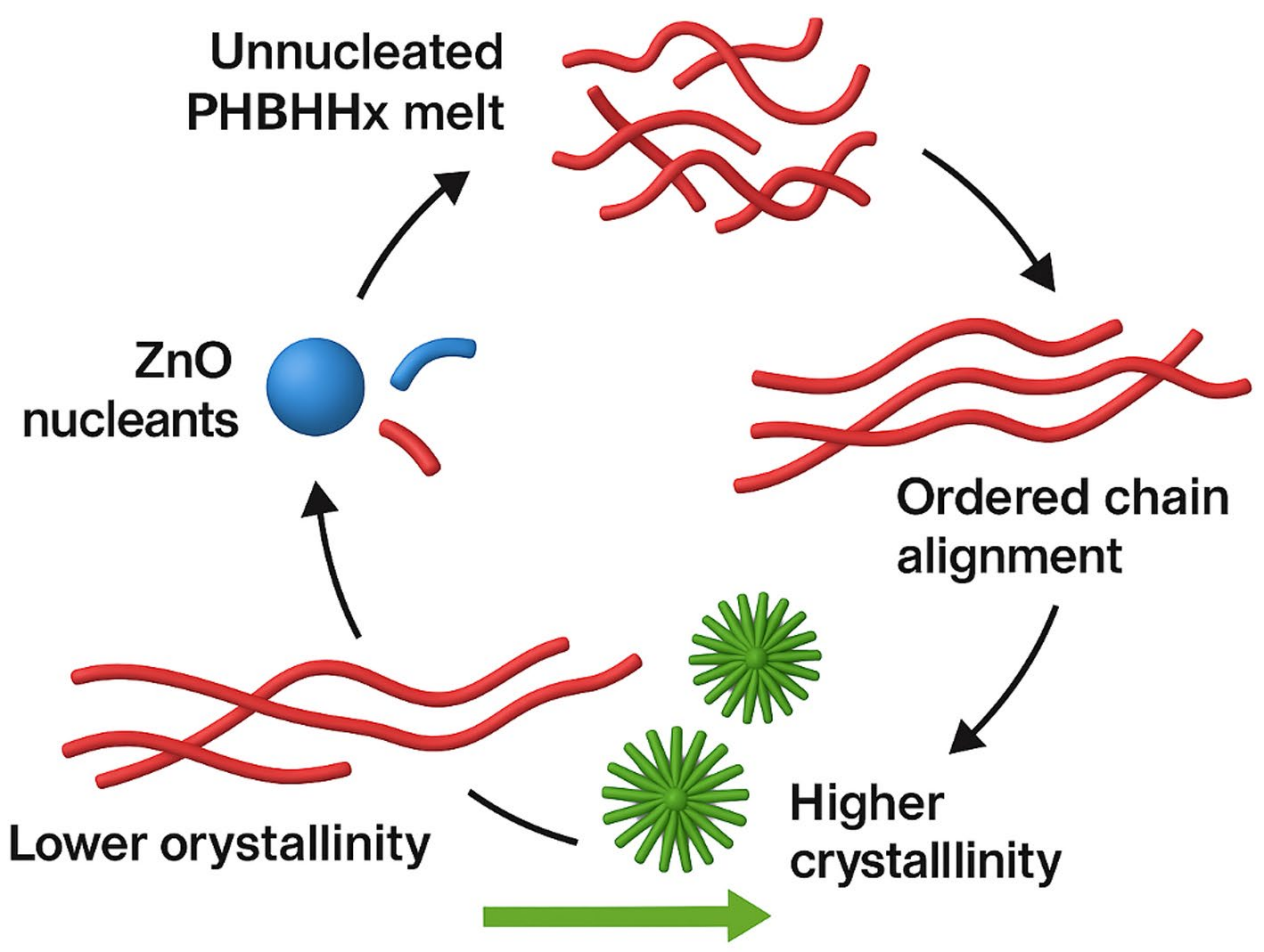
Overview of the crystallization pathway promoted by ZnO and its influence on PHBHHx film morphology.

PHBHHx-ZnO films prepared with controlled particle types show tighter morphology and more uniform dispersion, which supports modulus gains and lower gas transmission (22, 27). Uniform dispersion also reduces haze and supports optical clarity in thin films while maintaining antimicrobial and UV-shielding function at modest ZnO loadings. Crystallinity increases of 3–12% and spherulite refinement across PHAs and PLA confirm the consistency of ZnO-driven nucleation (22, 27). Figure 3 depicts the crystallization pathway promoted by ZnO and its influence on PHBHHx film morphology.

### 2.4 Synergy with natural bioactive compounds

ZnO pairs effectively with essential oils and phenolic extracts, delivering [combined] antimicrobial and antioxidant action with stable performance under refrigeration (28, 40). Studies in PLA, PHBV, cellulose and PVA matrices report similar synergistic behavior, confirming broad compatibility between ZnO and plant-derived antimicrobials (27, 33, 39). Synergistic effects appear consistently under chilled conditions, where microbial and oxidative pathways progress slowly but remain active (28, 40).

Hybrid ZnO structures maintain dispersion and functional stability in biopolymer matrices (27). PHBHHx provides a suitable matrix for these hybrid systems due to its flexibility, moderate crystallinity, and stable interactions with both ZnO and natural compounds (18, 31, 32). These hybrid systems often show enhanced antioxidant capacity and improved microbial suppression compared with either component alone.

### 2.5 Relevance for food-packaging systems

PHBHHx offers a compatible, flexible matrix that disperses ZnO well and holds activity at low particle fractions. The combined system brings antimicrobial control, effective UV shielding, and nucleation-driven barrier gains into one film architecture, aligning with shelf-life extension goals across fish, meat, dairy, bakery, and fresh produce (26, 31, 32). Mechanical and barrier gains appear consistently across PHBV, PLA, starch, cellulose, and chitosan systems, strengthening expectations for comparable behavior in PHBHHx (27, 28, 33). Practical builds rely on moderate loadings, stable dispersion, and multilayer placement to balance activity with clarity and end-of-life considerations (22, 38). Multilayer formats also help confine ZnO within a functional active layer, supporting clarity and reducing migration risk.

Current regulatory assessments emphasize that ZnO nanoparticles are not expected to migrate in particulate form under typical use conditions; regulatory focus is placed on ionic Zn^2+^ migration, which must comply with the EU-specific migration limit of 5 mg/kg food. EFSA additionally advises considering total dietary zinc exposure when designing active-packaging systems (EU 10/2011; 60) (22, 38).

A short overview of ZnO nanoparticle functions across biodegradable matrices clarifies the breadth of activity relevant to PHBHHx-ZnO films. The main roles span antimicrobial control, UV shielding, crystallinity development, antioxidant support, compatibility with natural bioactive compounds, optical behavior, and mechanical or thermal stability, with migration-safety requirements included (22). Table 1 summarizes these functional themes with practical packaging relevance.

**Table 1:** Functional contributions of ZnO nanoparticles to biodegradable food-packaging matrices.

ZnO contribution	Packaging relevance	Representative sources	References
Antimicrobial activity	ROS generation, Zn^2+^ release, cell contact injury	Reduces spoilage organisms and total viable counts under chilled storage	(16, 36)
UV blocking	Wide bandgap absorption (UVA/UVB)	Protects light-sensitive foods and delays photo-oxidation	(23, 38)
Nucleation/crystallinity	Seeds ordered chain growth; increases crystallinity	Enhances stiffness and lowers oxygen transmission through tortuosity effects	(26, 28)
Antioxidant support	Photocatalytic/ROS quenching in hybrids	Slows lipid oxidation in chilled foods	(27, 40)
Synergy with natural bioactive compounds	Stabilizes phenolics, essential oils	Delivers joint antimicrobial and antioxidant responses	(33, 39)
Optical clarity (when dispersed)	Uniform particle distribution	Maintains transparency and low haze in thin films	(22)
Thermal/mechanical reinforcement	Raises decomposition temperature, storage modulus	Supports processability and active-layer durability	(41)
Migration safety	Non-migration in nanoform; only ionic Zn^2+^ assessed	Complies with EU SML of 5 mg/kg food; EFSA advises considering total dietary zinc exposure	EU 10/2011. (60)

## 3 PHBHHx: structure, properties, and relevance

### 3.1 Chemical structure and crystallinity

PHBHHx is a PHA copolymer with 3-hydroxyhexanoate units that interrupt chain regularity. These units reduce crystallinity and increase flexibility relative to PHB and PHBV (18–20). Infrared and thermal analyses indicate looser packing and slower spherulite growth as 3-hydroxyhexanoate content rises (18, 21). The matrix bends without early fracture and accepts nanoparticle nucleants evenly (26). PHBHHx maintains semicrystalline behavior at packaging-grade compositions, which supports stable film formation (18, 19).

Melt-processed PHBHHx-ZnO films retain flexibility and show crystallinity around 53–56%, confirming that ZnO does not disrupt the semicrystalline framework (22).

**Figure 4:**
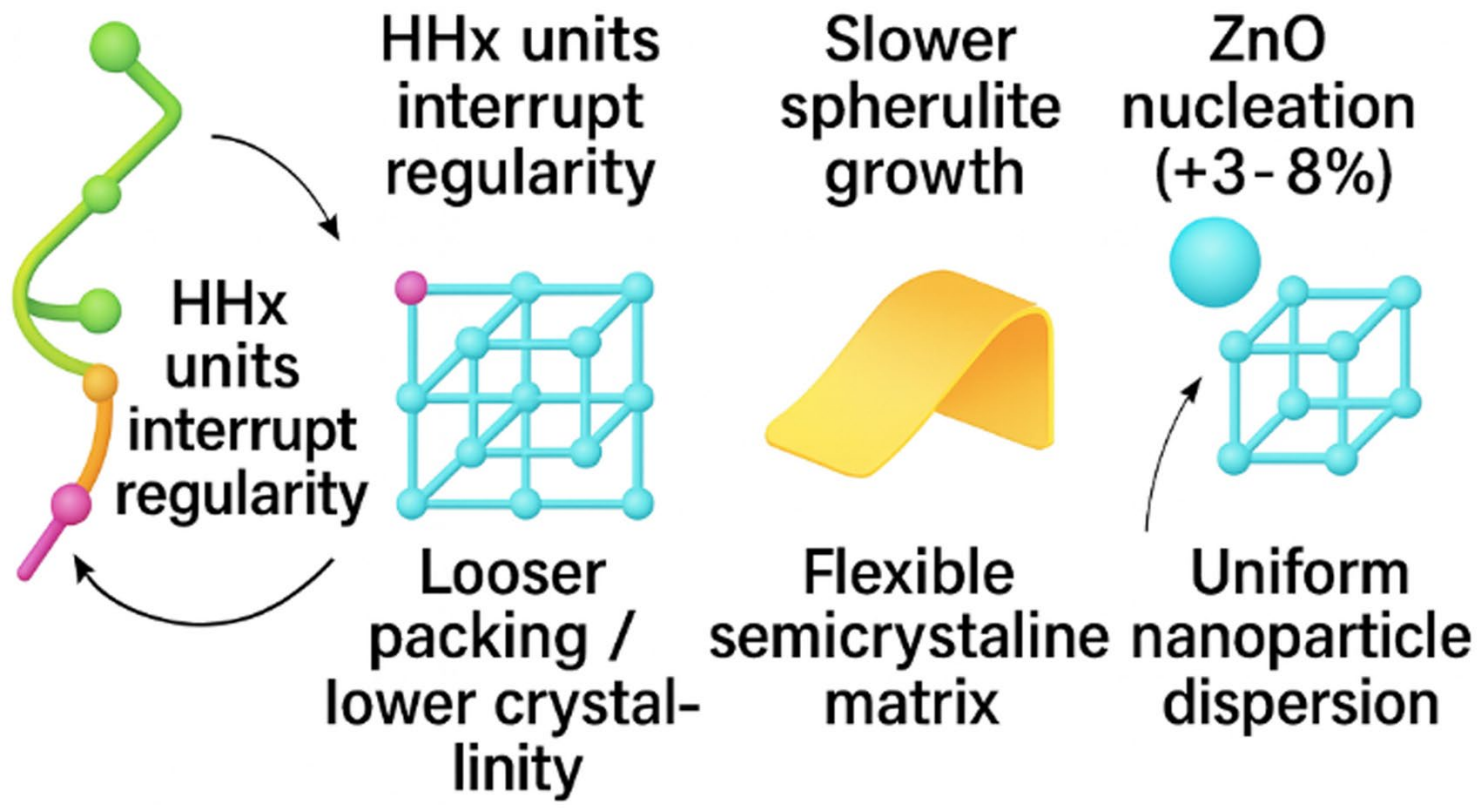
Structural features of PHBHHx and the influence of 3-hydroxyhexanoate units on crystallinity and multifunctional behavior.

Crystallinity increases of around 3–8% have been reported at low ZnO loadings, consistent with nucleation trends observed in PLA, PHBV, and cellulose matrices. PHBHHx contains 3-hydroxyhexanoate units that interrupt chain regularity, reduce crystallinity, and increase flexibility relative to PHB and PHBV. These structural features slow spherulite growth, loosen chain packing, and support uniform nanoparticle dispersion during melt processing. The resulting semicrystalline framework maintains film stability while enabling multifunctional performance when ZnO is incorporated (27, 33, 39). Figure 4 illustrates the structural characteristics of PHBHHx and shows how 3-hydroxyhexanoate units influence crystallinity and film behavior.

### 3.2 Thermal and mechanical behavior

PHBHHx tolerates processing temperatures better than PHB because lower crystallinity delays brittleness and reduces melt-fracture risk (8, 19). Mechanical data place PHBHHx between PHB and toughened blends: elongation at break exceeds that of PHB or PHBV, and tensile strength rises after nucleation or filler addition (18, 26, 41). Fiber-spinning and film studies report stable modulus and wider toughness windows under controlled cooling (19, 42). Thermal-stability gains support repeated heating cycles during melt processing (8, 42).

**Figure 5:**
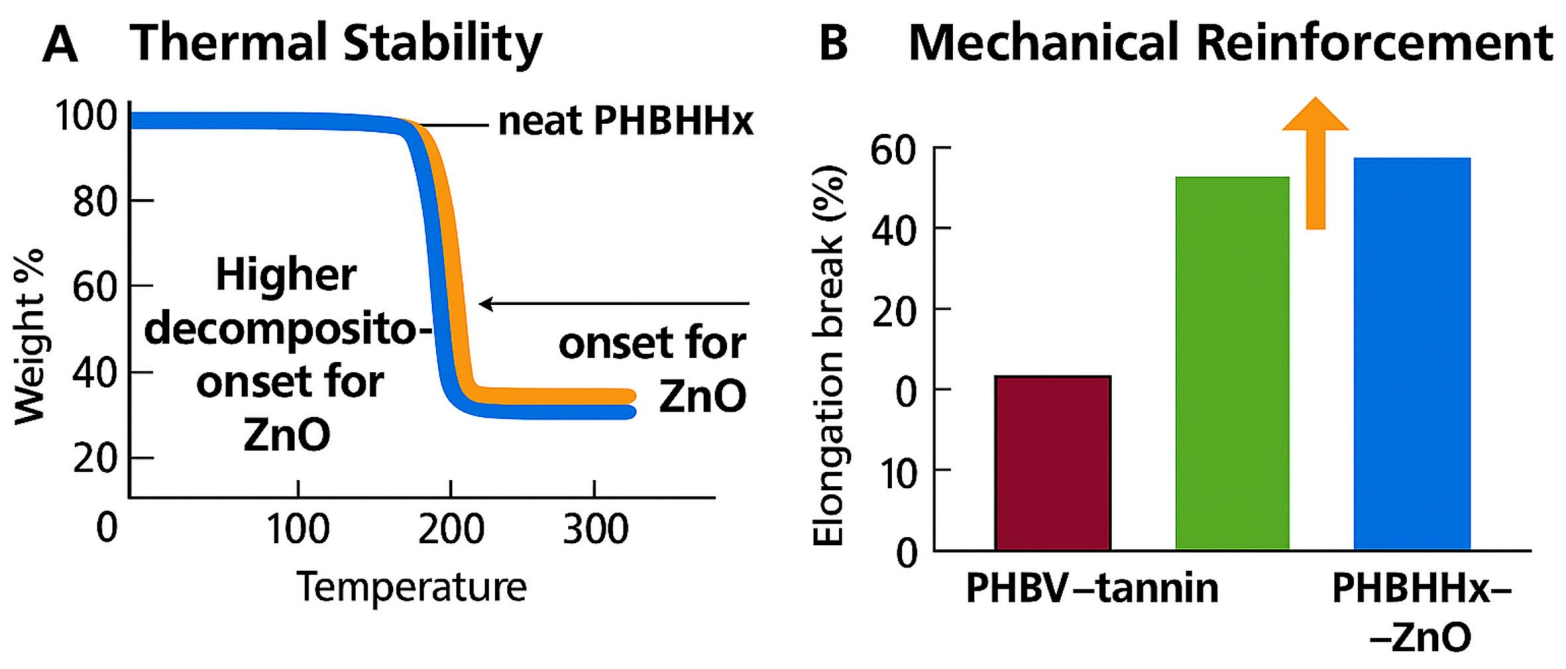
**(A)** Thermal stability of neat PHBHHx and PHBHHx–ZnO showing a higher decomposition‑onset temperature for ZnO‑reinforced films. **(B)** Mechanical reinforcement indicated by elongation‑at‑break comparison between PHBV–tannin and PHBHHx–ZnO.

PHBHHx-ZnO films show rheological and mechanical adjustments linked to nanoparticle type while maintaining flexibility (22). ZnO loadings of 1–5% typically increase tensile modulus by around 10–25% and raise decomposition-onset temperature by around 10–20 °C without compromising elongation at break (26, 41). PHBHHx exhibits improved thermal tolerance and mechanical flexibility compared with PHB, and ZnO addition further adjusts modulus, strength, and toughness through nucleation and interfacial interactions (8, 22). Reinforcement trends depend on particle type, dispersion quality, and cooling conditions, with well-dispersed ZnO supporting stable modulus and reduced brittleness (22, 26, 41). Comparative PHBV-tannin systems show increasing brittleness at higher tannin loadings, with particle sizes shifting from around 0.3–0.5 μm to around 1.3 μm (43). Figure 5 maps the thermal-stability and mechanical-reinforcement trends observed in PHBHHx-ZnO films.

### 3.3 Barrier properties and moisture sensitivity

PHBHHx provides a moderate oxygen barrier for a semicrystalline polyester, with OTR values commonly around 80–120 cc/m^2^·day at 23 °C and 50% RH. PHB shows stronger resistance at around 20–40 cc/m^2^·day, PLA exhibits weaker barrier behavior at around 150–300 cc/m^2^·day, and PHBV provides one of the lowest OTR values among PHAs at around 10–20 cc/m^2^·day. Crystallinity and diffusion tortuosity remain the main determinants of oxygen transport (23, 38). Moisture barrier remains moderate due to hydrophobic chains and limited water uptake, although multilayers or coatings improve performance for high-humidity foods (17, 44).

**Figure 6:**
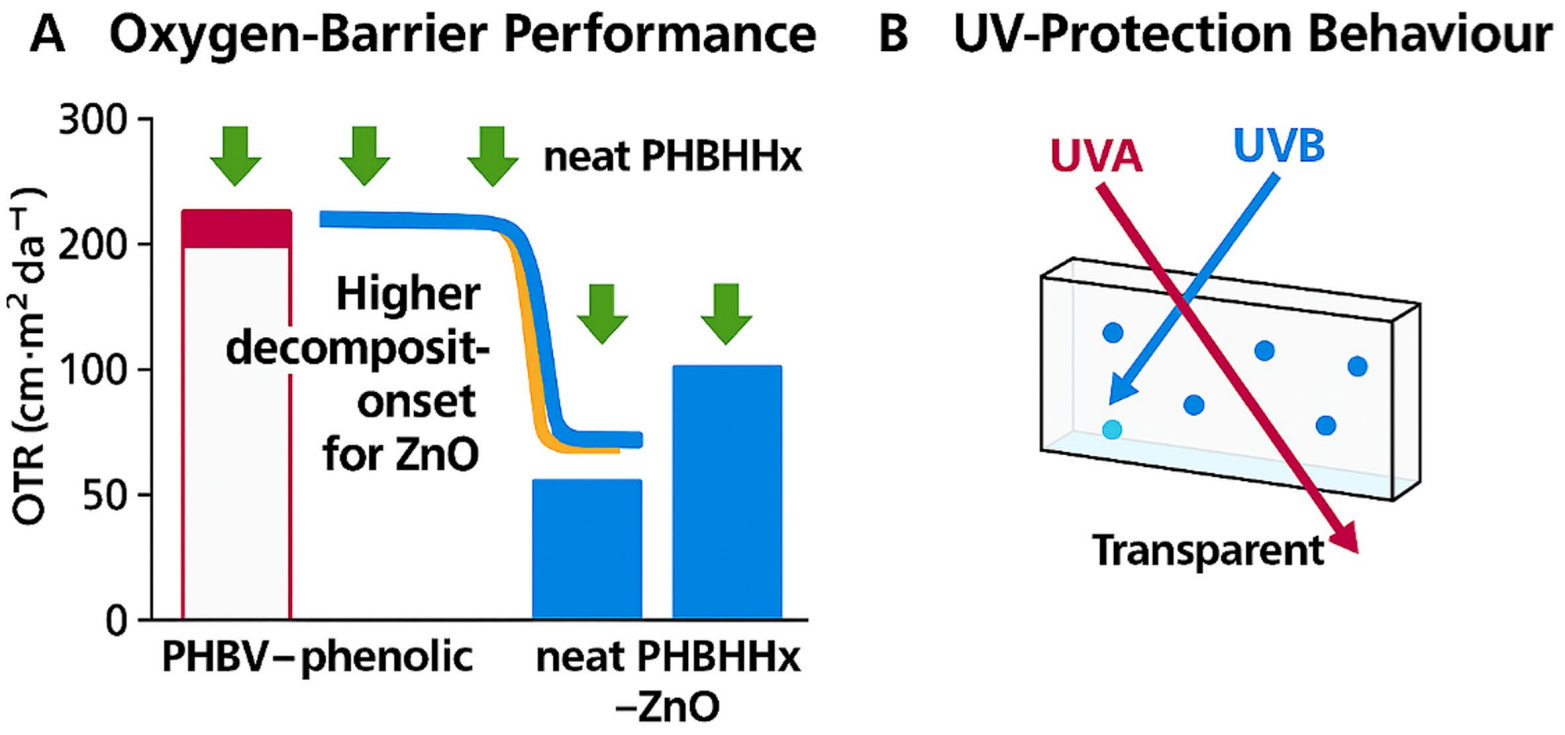
**(A)** Oxygen‑barrier performance of PHBV–phenolic, neat PHBHHx, and PHBHHx–ZnO, showing reduced OTR and higher decomposition‑onset associated with ZnO reinforcement. **(B)** UV‑protection behaviour of ZnO‑containing films, illustrating UVA blocking and modified UVB transmission through the transparent matrix.

Barrier improvements remain consistent across films and coated structures when ZnO dispersion is uniform (23, 38). PHBHHx-ZnO films show measurable UV and oxygen-barrier gains at 1–5% ZnO, with transparency retained at low loadings (22). Oxygen-transmission reductions of around 15–40% and UV-transmittance reductions of around 60–95% have been reported at low ZnO loadings. ZnO nanoparticles enhance oxygen-barrier behavior by increasing crystallinity and creating tortuous diffusion pathways, while strong UVA/UVB absorption provides active UV shielding. These effects appear at modest ZnO loadings and remain compatible with transparency when dispersion is uniform (22, 23, 38). Figure 6 highlights the oxygen-barrier gains and UV-protection behavior of PHBHHx-ZnO films.

### 3.4 Processing windows and morphology control

PHBHHx processes by extrusion, blown film, casting, and fiber spinning with a wider latitude than PHB or PHBV. Nucleants raise crystallization rate and tighten spherulites, which stabilize modulus and lower gas permeability (22, 26). Processing studies recommend moderate shear and controlled cooling to avoid in-line degradation; blends, chain extenders, or targeted nanoparticle placement extend the window further (42, 45). Reduced crystallinity lowers melt viscosity and improves stability during extrusion (8, 19). Centrifugal fiber spinning of PHBHHx produces tunable microfibers and annealed top layers suitable for multilayer structures (19).

**Figure 7:**
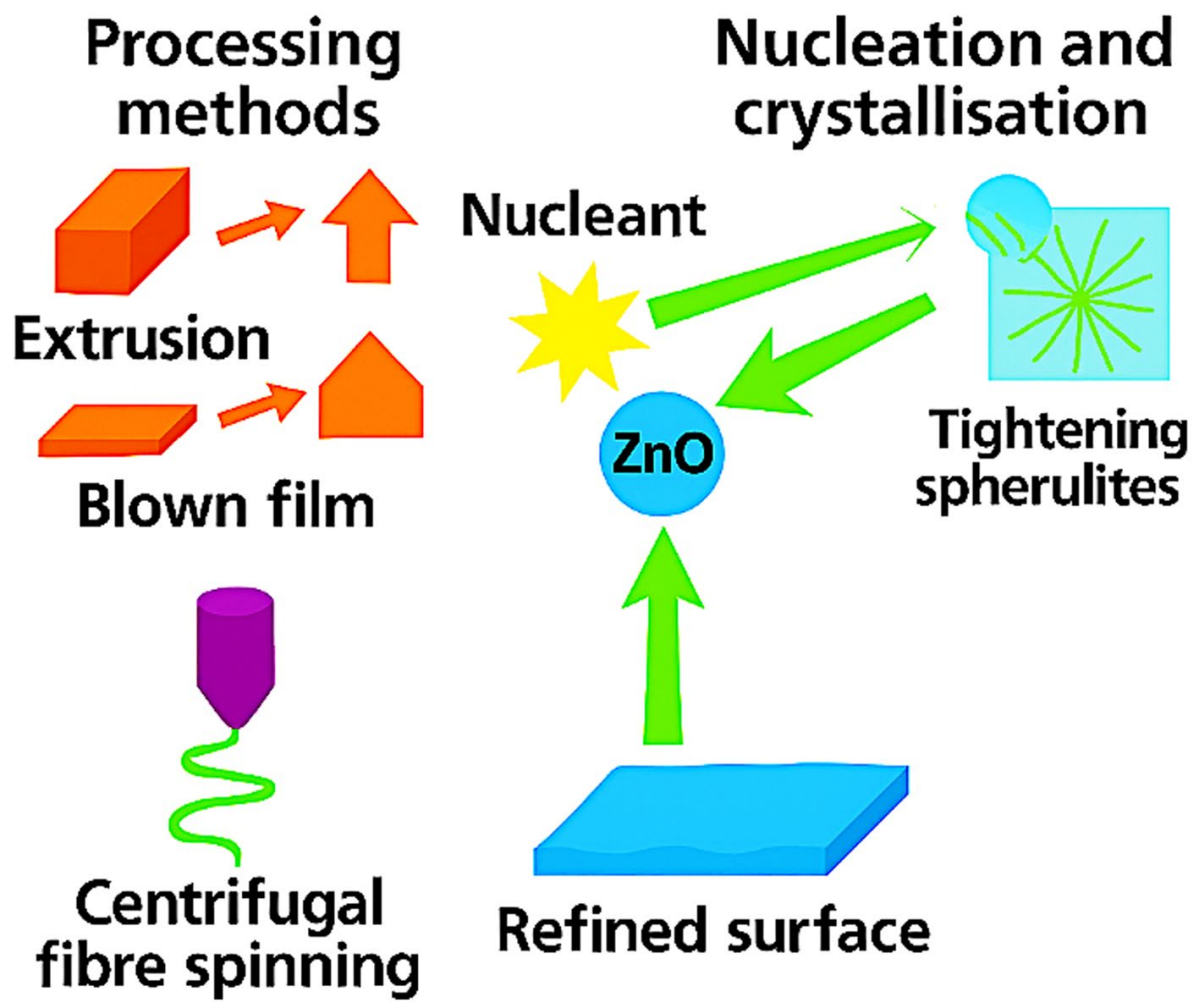
Morphology refinement and crystallization control in PHBHHx-ZnO films.

ZnO-reinforced PHBHHx films show refined spherulites, smoother fracture surfaces, and more uniform nanoparticle dispersion, which together support mechanical stability and optical clarity. ZnO nanoparticles act as efficient nucleants in PHBHHx, refining spherulite size, tightening morphology, and promoting more uniform crystallization during cooling. These effects stabilize modulus, reduce gas permeability, and support smoother fracture surfaces in processed films. Controlled dispersion further enhances optical clarity and mechanical stability across extrusion, casting, and fiber-spinning formats (22, 26). Figure 7 presents the morphology refinement and crystallization control achieved in PHBHHx-ZnO systems.

### 3.5 Biodegradation and end-of-life

PHBHHx degrades under industrial composting and proceeds in soil and marine environments through microbial esterases and depolymerases (7, 8). Low ZnO loadings do not hinder biodegradation and remain immobilized within crystalline regions during early stages (31, 32). Multilayer formats that confine ZnO to functional strata support safer end-of-life performance while preserving activity in use (22). PHBHHx reinforced with natural compounds maintains compatibility with microbial breakdown pathways (18). Biodegradation rates remain comparable to PHBV and PLA under similar conditions, supporting integration into circular-economy strategies (7, 8).

**Figure 8:**
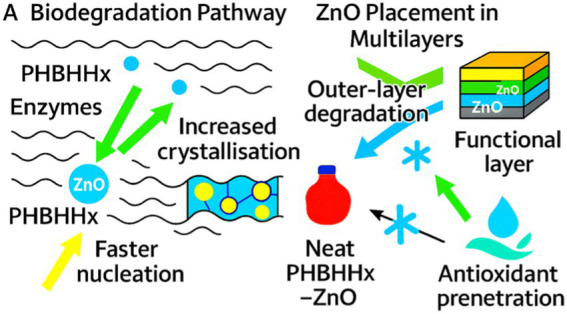
Biodegradation pathways and ZnO placement effects in multilayer PHBHHx structures.

ZnO does not migrate in nanoform during degradation; only ionic Zn2 + release is relevant and remains within regulatory limits under composting conditions (7, 8). Functional-layer placement helps confine nanoparticles, supporting safe end-of-life behavior while maintaining activity in use. Biodegradation proceeds through enzymatic cleavage of ester bonds in compost, soil, and marine environments, with ZnO remaining immobilized in crystalline regions during early stages (31, 32). Figure 8 highlights the biodegradation pathways of PHBHHx films and shows how ZnO placement influences early-stage behavior.

### 3.6 Why PHBHHx fits food-packaging tasks

PHBHHx balances flexibility, thermal stability, and barrier control better than many single-polymer bioplastics. The matrix disperses ZnO and plant-derived bioactive compounds uniformly, which enables antimicrobial, UV-shielding, and oxidative-stability functions within thin films (27, 28). PHBHHx-ZnO films maintain crystallinity around 53–56%, which supports mechanical integrity, optical clarity, and consistent barrier behavior during chilled storage (22). These traits suit fish, meat, dairy, bakery, and fresh-produce packs that need shelf-life gains without persistent plastic residues (17, 24). Real-food studies in seafood and beverages report microbial reductions and slower oxidation at modest ZnO loadings, confirming practical relevance for high-risk foods (11, 12).

PHBHHx also supports multilayer and coated formats that incorporate a designated active layer, allowing functional components such as ZnO to deliver antimicrobial and UV-protective behavior while keeping migration within safe limits (22, 31, 32). PHBHHx consistently shows the widest processing latitude among the compared matrices, the most stable ZnO dispersion, and the most balanced combination of flexibility, crystallinity, and barrier behavior. These traits underpin its suitability for active-packaging formats that require mechanical stability, optical clarity, and controlled migration. PHBHHx offers the most balanced combination of flexibility, crystallinity, dispersion stability, and active-function compatibility among the listed matrices (18, 19, 22). Table 2 compares the structural traits, mechanical responses, and ZnO-driven functional gains across common biodegradable matrices to contextualize PHBHHx performance.

**Table 2:** Structural and functional comparison of ZnO-reinforced biopolymer matrices.

Matrix	Crystallinity and mechanical traits	ZnO-related behavior	References
PHBHHx	Semicrystalline; 53–56%; flexible; wide processing range	Uniform dispersion; strong UV and O₂ barrier; low haze	(18, 19, 22)
PHB	High crystallinity; low flexibility; narrow processing range	Moderate dispersion; strong UV; moderate O₂ barrier	(8, 18)
PHBV	Moderate crystallinity; brittle at low humidity	Stable dispersion; strong O₂ barrier; moderate UV	(6, 43, 46)
PLA	Moderate crystallinity; low elongation; shear-sensitive	Moderate dispersion; strong UV; moderate barrier gains	(27, 33)
Starch	Low crystallinity; weak strength; moisture-sensitive	Strong barrier gains; moderate UV; limited dispersion stability	(33, 39)
Cellulose	Moderate crystallinity; rigid; limited flexibility	Strong UV and O₂ barrier; moderate dispersion; low haze	(28, 46)

## 4 PHBHHx-ZnO bionanocomposites: structure–property relationships

### 4.1 Influence of ZnO on PHBHHx crystallinity and morphology

PHBHHx displays moderate crystallinity because 3-hydroxyhexanoate units reduce chain regularity and increase flexibility (18–20). Neat PHBHHx typically shows crystallinity around 50%, and the incorporation of 3% ZnO increases this to approximately 53–56%, reflecting more ordered chain packing and faster crystallization (22, 41). Biopolymer matrices such as PHAs, PLA, starch, and cellulose consistently demonstrate similar nucleation effects, confirming the broad nucleating behavior of ZnO (6, 27, 28, 33, 39, 46). Recent work on PHBH and PHBHV copolymers also shows crystallinity increases of around 3–10% at low ZnO loadings, paralleling PHBHHx trends and confirming consistent nucleation across PHA families (13, 21). ZnO.

PHBHHx-ZnO materials with higher crystallinity exhibit improved mechanical strength, reduced gas permeability, and enhanced thermal stability (26, 28). ZnO nanoparticles act as efficient nucleants, refining spherulite size, tightening morphology, and promoting more uniform crystallization during cooling (22, 41). Excessive ZnO loading at 6% or higher leads to agglomeration and diminished thermal resistance, reflecting dispersion limits observed in other ZnO-reinforced biopolymers (23, 41).

**Figure 9:**
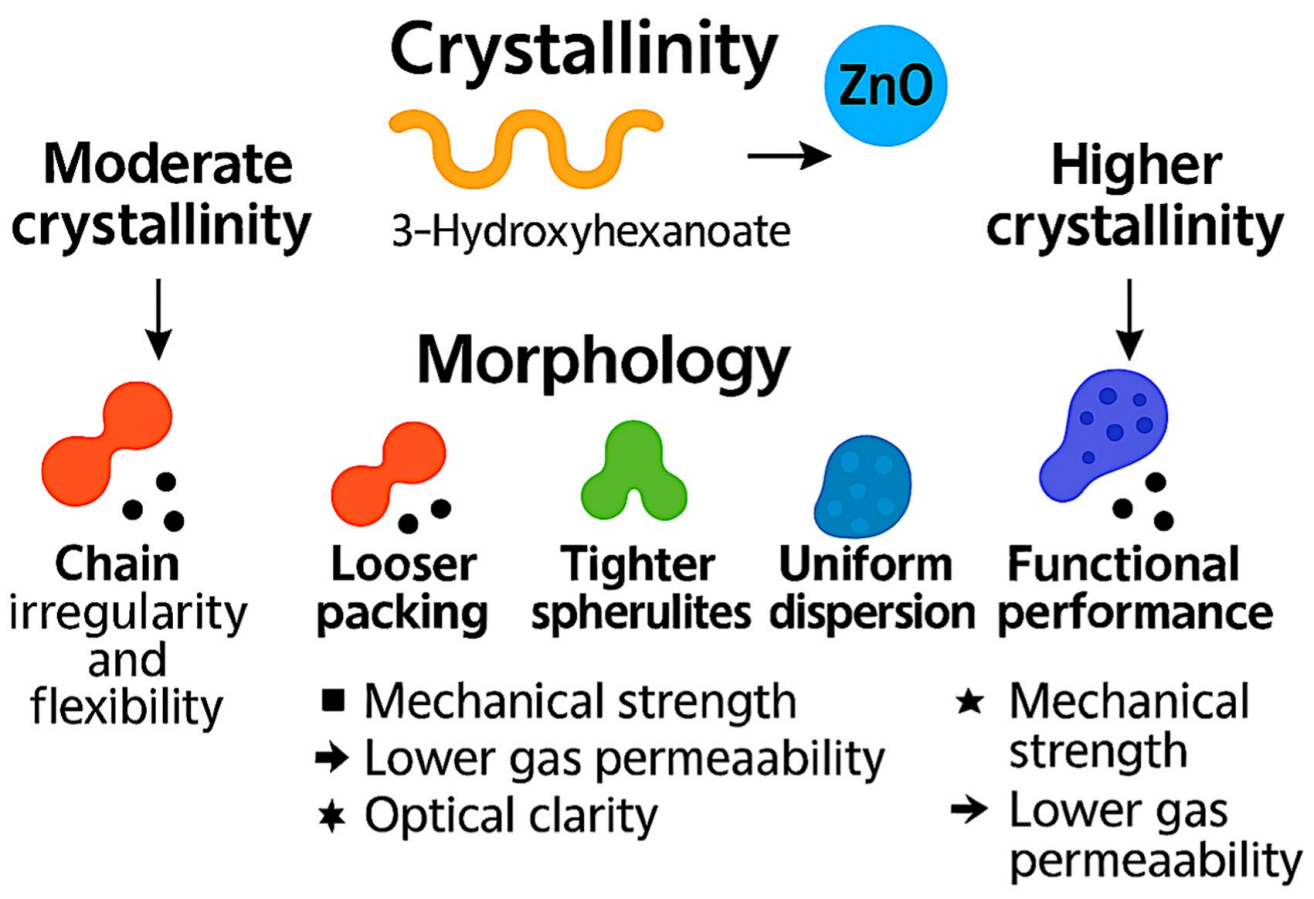
Structure–property relationships of PHBHHx-ZnO bionanocomposites.

Surface-modified or hybrid ZnO types display stronger dispersion and more uniform interfacial interactions, stabilizing morphology across films and fibers (23, 29). PHBHHx-ZnO films with uniform dispersion also show better optical clarity and lower haze (22, 27). Multilayer PHBHHx structures containing ZnO show tighter spherulites and more stable crystalline domains, confirming that ZnO supports morphology control across laminated builds (35). Figure 9 illustrates the structure–property relationships that govern crystallinity, morphology, and functional performance in PHBHHx-ZnO systems.

### 4.2 Mechanical performance of PHBHHx-ZnO systems

PHBHHx exhibits higher flexibility than PHB because the 3-hydroxyhexanoate units disrupt crystallinity, while tensile strength and modulus remain moderate (18–20, 41). PHBHHx-ZnO nanocomposites demonstrate higher tensile strength and Young’s modulus due to stronger interfacial adhesion and restricted chain mobility (22, 41). Biodegradable matrices such as PHAs, PLA, starch, and cellulose show comparable ZnO-driven reinforcement, confirming a general mechanical-enhancement role for ZnO across biopolymer families (6, 27, 28, 33, 46). Electrospun and fiber-spun PHBHHx scaffolds also show modulus increases of around 10–20% and toughness gains when doped with ZnO, confirming reinforcement across multiple processing formats (19, 22).

Biodegradable films containing low ZnO contents maintain food-contact safety while delivering measurable mechanical gains (11, 31). Excessive ZnO loading reduces elongation at break due to particle agglomeration and local stress concentration (11, 31). PHBHHx tolerates moderate ZnO contents better than more brittle PHB, supporting wider composition windows for active-packaging applications (18, 19). PHBHHx-ZnO nanocomposites show higher tensile strength and modulus through stronger polymer-nanoparticle adhesion and restricted chain mobility, while maintaining flexibility at low ZnO loadings (22, 41).

### 4.3 Barrier properties and moisture control

PHBHHx films provide moderate oxygen and moisture barrier performance, consistent with their semicrystalline structure (4, 24). PHBHHx-ZnO films show lower oxygen-transmission rates because increased crystallinity and more tortuous diffusion pathways restrict gas transport (23, 38). Similar oxygen-barrier enhancements have been documented in PHBV-ZnO, PLA-ZnO, and cellulose-ZnO matrices, confirming a consistent tortuosity-driven barrier mechanism across biodegradable polymers (31, 33, 46). Food categories such as fish, meat, dairy, and fresh produce benefit from these barrier gains, as oxidative deterioration progresses rapidly in these products (4, 24).

PHBHHx-ZnO structures also display modest improvements in moisture barrier due to reduced free volume and stronger polymer-nanoparticle interactions (17, 44). Hydrophilic foods that require stronger moisture control still rely on multilayer builds or coated formats, in line with observations for PLA, PHBV, and starch systems (17, 44, 61) (Liu et al., 2022). PHBHHx-ZnO films further show reduced UV transmission because ZnO absorbs strongly in the UVA and UVB regions, limiting photo-oxidation, pigment loss, and nutrient degradation (23, 38).

ZnO increases crystallinity and creates tortuous diffusion pathways that lower oxygen-transmission rates, while strong UVA/UVB absorption reduces photo-oxidation and pigment loss. Moisture-barrier improvements arise from reduced free volume and stronger polymer-nanoparticle interactions. These enhancements support active preservation in oxygen- and light-sensitive foods (17, 23, 38, 44).

### 4.4 Thermal stability and processing behavior

PHBHHx materials display moderate thermal stability, with degradation onset close to typical processing temperatures (8, 19). Neat PHBHHx typically begins degrading at around 250–260 °C, whereas adding 3% ZnO increases this onset by approximately 10–20 °C and raises Tmax by around 15–25 °C, reflecting restricted chain mobility and a more stabilized matrix (26, 41). PHBHHx-ZnO nanocomposites also show lower thermal-oxidative degradation than neat PHBHHx, supporting repeated processing cycles and industrial-scale manufacture of films and coatings (19, 42). Biodegradable matrices such as PHBV, PLA, and cellulose show similar ZnO-induced thermal improvements, confirming a general stabilizing role for ZnO across biopolymer families (31, 32).

Processed PHBHHx-ZnO materials maintain molecular weight more effectively, with less chain scission and lower brittleness in final products (8, 31). Hybrid ZnO formulations, including doped, ligand-stabilized, and core-shell types, further improve thermal stability in PHBHHx stacks and reduce oxidative degradation during processing (5, 29). ZnO raises decomposition-onset temperatures, reduces thermal-oxidative degradation, and stabilizes molecular weight during processing, supporting repeated heating cycles and industrial-scale manufacture of films, fibers, and coatings. Hybrid ZnO structures further enhance thermal stability by limiting chain scission and oxidative pathways (5, 29, 31, 41).

### 4.5 Optical behavior and UV protection

Neat PHBHHx films show moderate transparency and limited intrinsic UV protection (23, 38). PHBHHx-ZnO films exhibit lower UV transmittance because ZnO provides strong absorption in UVA and UVB regions (23, 38). ZnO-coated PHBHHx and ZnO-sprayed PET structures also show reinforced UVA and UVB attenuation, confirming that UV shielding is governed by ZnO rather than matrix chemistry (38). Biopolymer systems based on PLA, PHBV, cellulose, and starch reveal comparable UV-blocking behavior when loaded with ZnO, indicating that optical protection arises primarily from the nanofiller rather than the matrix chemistry (27, 28, 33, 46).

PHBHHx-ZnO films with surface-modified ZnO particles show better clarity and reduced haze compared with systems containing unmodified particles, supporting transparent-packaging uses (22, 27). Smart-packaging studies identify PHBHHx-ZnO as a suitable platform for UV-responsive sensing layers and freshness-indicator films due to its stable UV absorption and optical clarity (4, 35). PHBHHx-ZnO films show reduced UV transmittance because ZnO strongly absorbs UVA and UVB radiation, while uniform dispersion maintains transparency and low haze. Surface-modified ZnO particles further improve clarity, supporting transparent packaging and UV-responsive sensing applications (4, 22, 23, 27, 35, 38).

### 4.6 UV protection

PHBHHx-ZnO films show strong UV-blocking behavior because ZnO absorbs efficiently in UVA and UVB regions, reducing photo-oxidation, pigment loss, and nutrient degradation (23, 38). PLA, PHBV, cellulose, and starch systems show similar UV-shielding when ZnO is uniformly dispersed, confirming that UV protection arises from the nanofiller rather than the polymer matrix (27, 28, 33, 46). PHBHHx-ZnO films maintain stable clarity at low ZnO loadings, confirming suitability for visible-product displays while delivering active UV protection. Smart-packaging reviews identify ZnO as a core UV-shielding component in multifunctional films and highlight its relevance for UV-responsive sensing layers (5, 35).

**Figure 10:**
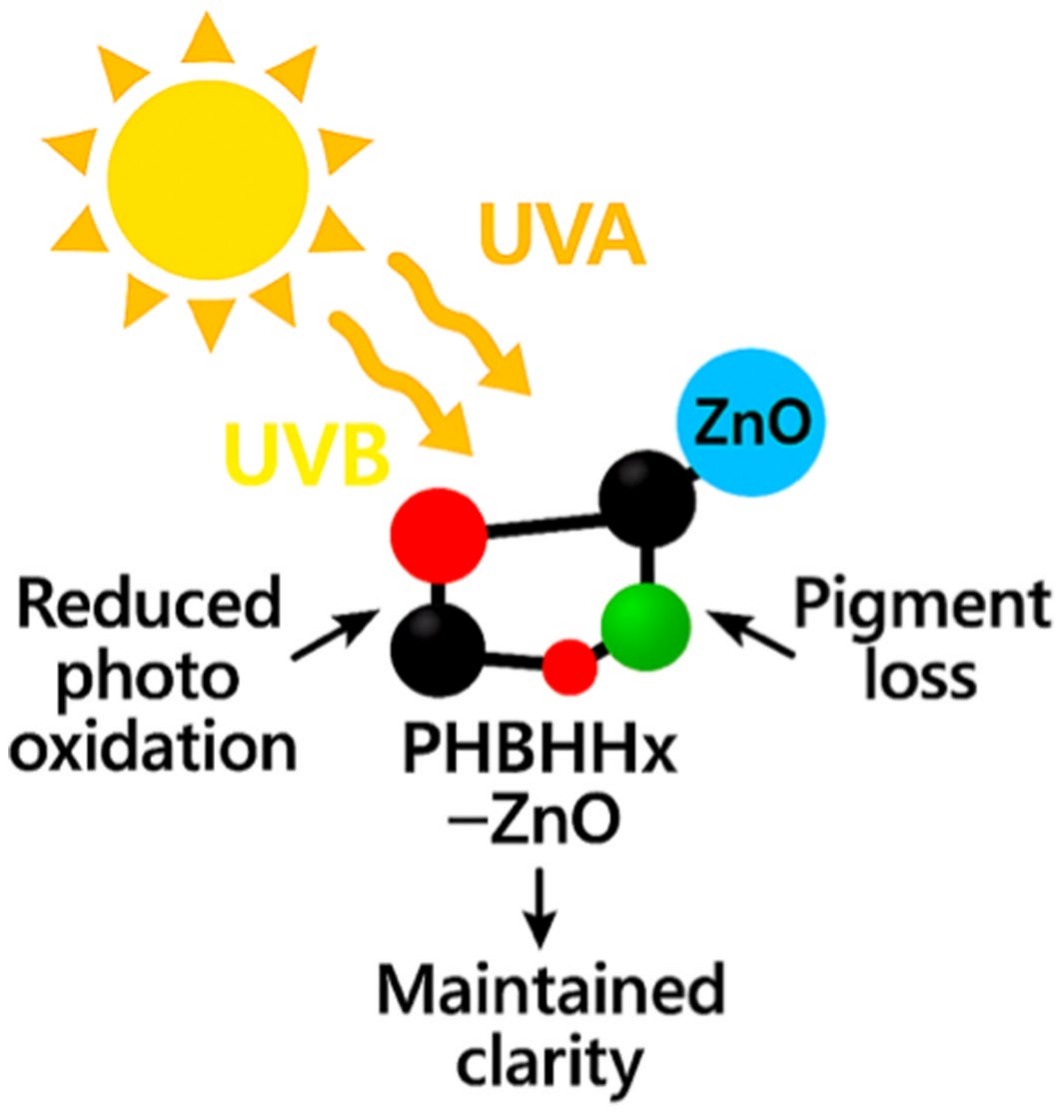
UV-blocking behavior and optical-clarity performance of PHBHHx-ZnO films.

ZnO provides strong UVA and UVB absorption, enabling PHBHHx-ZnO films to block light-induced degradation, pigment loss, and nutrient deterioration in sensitive foods. Comparable UV-shielding behavior appears in PLA-ZnO, PHBV-ZnO, cellulose-ZnO, and starch-ZnO matrices when ZnO is uniformly dispersed, confirming that optical protection arises from the nanofiller rather than the polymer chemistry. Surface-modified ZnO particles further improve clarity and reduce haze across these systems, supporting transparent-packaging formats and UV-responsive sensing applications (5, 22, 23, 27, 35, 38). Figure 10 presents the UV-absorption patterns and optical-clarity behavior of PHBHHx-ZnO films.

## 5 Antimicrobial and antioxidant behavior of PHBHHx-ZnO systems

### 5.1 Antimicrobial performance against spoilage organisms and pathogens

PHBHHx-ZnO systems show strong antimicrobial activity due to multiple mechanisms that operate simultaneously. Early work demonstrated that ZnO generates reactive oxygen species that disrupt membrane integrity and damage intracellular components (12, 36). Later studies confirmed that Zn^2+^ ions destabilize cell walls, interfere with metabolic pathways, and intensify membrane damage through direct nanoparticle-cell contact (30, 37). Trials in fish, meat, and dairy systems consistently report around 1–2 log CFU/g microbial reductions during refrigerated storage when ZnO is incorporated into biodegradable films (11, 16, 25). PHBHHx matrices support uniform nanoparticle dispersion, which strengthens antimicrobial performance compared with more brittle matrices such as PHB or PLA (18, 19).

Comparative studies across PHBV, PLA, starch, alginate, and cellulose systems show similar antimicrobial trends, confirming the broad relevance of ZnO for biodegradable packaging (6, 27, 28, 33, 46). Additional work on PHBH-based films demonstrates comparable ZnO-driven antimicrobial reductions, confirming cross-matrix generality (13, 21, 54). Smart-packaging reviews further identify ZnO as a core antimicrobial component in multifunctional films (5, 35, 54).

### 5.2 Synergistic effects with natural bioactive compounds

PHBHHx-ZnO systems show stronger antimicrobial and antioxidant behavior when natural extracts, essential oils, or phenolic compounds are incorporated. Early studies demonstrated synergy between ZnO and essential oils in biodegradable matrices (33, 55). More recent work confirms similar effects with phenolic compounds and plant-derived antimicrobials (27, 39, 40). These combinations often produce around 1 log CFU/g additional microbial reduction beyond ZnO alone, reflecting complementary ROS-scavenging and membrane-disruption pathways.

Studies in fish, meat, and bakery products report lower microbial loads, slower lipid oxidation, and improved sensory quality during chilled storage when ZnO is combined with natural bioactive compounds (24, 27). ZnO-bioactive combinations behave consistently in PLA, PHBV, and cellulose matrices, confirming broad compatibility between ZnO and plant-derived antimicrobials (27, 28, 40, 55).

PHBHHx matrices support hybrid antimicrobial systems because their flexibility, moderate crystallinity, and stable interactions with ZnO and natural compounds enable uniform dispersion and controlled release (18, 31, 32). Recent reviews report similar synergies in bionanocomposites containing polyphenols, lignin nanoparticles, and green-synthesized ZnO, confirming the generality of these interactions (40, 47, 48).

ZnO-bioactive combinations enhance antimicrobial and antioxidant behavior through complementary mechanisms, and PHBHHx provides a stable, flexible matrix that supports these synergistic effects (18, 24, 27, 31–33, 39, 40, 47, 48).

### 5.3 Antioxidant behavior and oxidative-stability control

PHBHHx-ZnO systems show strong antioxidant behavior due to photocatalytic activity and radical-scavenging effects. Studies in fish, poultry, and fresh-produce systems report lower peroxide formation, slower pigment degradation, and reduced oxidative rancidity during refrigerated storage (4, 17, 24, 25). Comparative work in PLA, PHBV, and cellulose matrices shows similar oxidative-stability improvements, confirming the broad antioxidant role of ZnO (28, 33, 46). Natural phenolics and essential oils further strengthen antioxidant behavior through radical-scavenging and metal-chelating effects (27, 33, 39).

**Figure 11:**
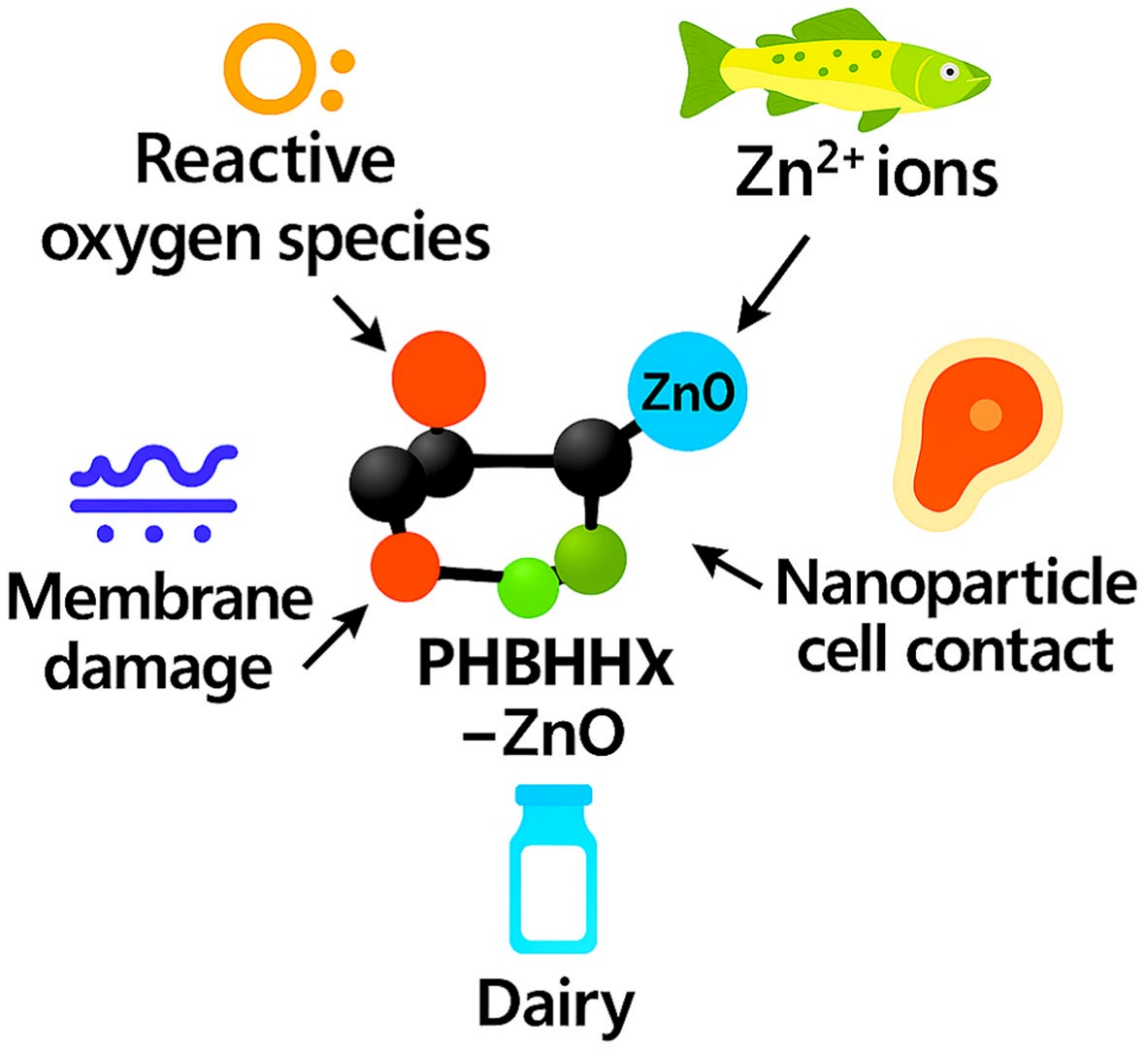
Antioxidant mechanisms and oxidative-stability control in PHBHHx-ZnO systems.

Hybrid ZnO structures stabilize oxidative behavior by maintaining dispersion and limiting agglomeration (23, 27). ZnO-polyphenol hybrids further amplify antioxidant activity in cellulose and PVA matrices, indicating potential for PHBHHx (27). Additional advances in doped and green-synthesized ZnO show enhanced oxidative stability across biodegradable systems (29, 40) (Figure 11).

ZnO provides photocatalytic radical scavenging, peroxide suppression, and pigment-stability support during chilled storage. Natural phenolics and essential oils add metal-chelating and radical-quenching functions, while hybrid ZnO-polyphenol structures amplify antioxidant activity through combined surface chemistry and dispersion stability. These coordinated mechanisms reduce oxidative rancidity and maintain color and nutrient integrity across fish, poultry, and fresh-produce systems (4, 17, 23–25, 27, 29, 33, 39, 40).

### 5.4 Shelf-life performance

PHBHHx-ZnO films extend shelf life in fish, meat, bakery, and fresh-produce systems because ZnO suppresses microbial growth, delays oxidation, and blocks UV-induced deterioration (11, 16, 56). Studies on gelatine-, EVA-, agar- and chitosan-based ZnO films show similar reductions in microbial load, slower lipid oxidation, and improved color retention, confirming that shelf-life gains arise from ZnO-enabled active functions rather than matrix chemistry (11, 33, 56, 58, 59).

PHBHHx-ZnO films combine antimicrobial activity, oxidative-stability support, and UV shielding, enabling extended preservation across high-risk foods. Reported extensions across ZnO-active systems range from 5 to 21 days depending on food type, storage conditions, and the presence of natural bioactives (11, 16, 33, 56, 58).

**Figure 12:**
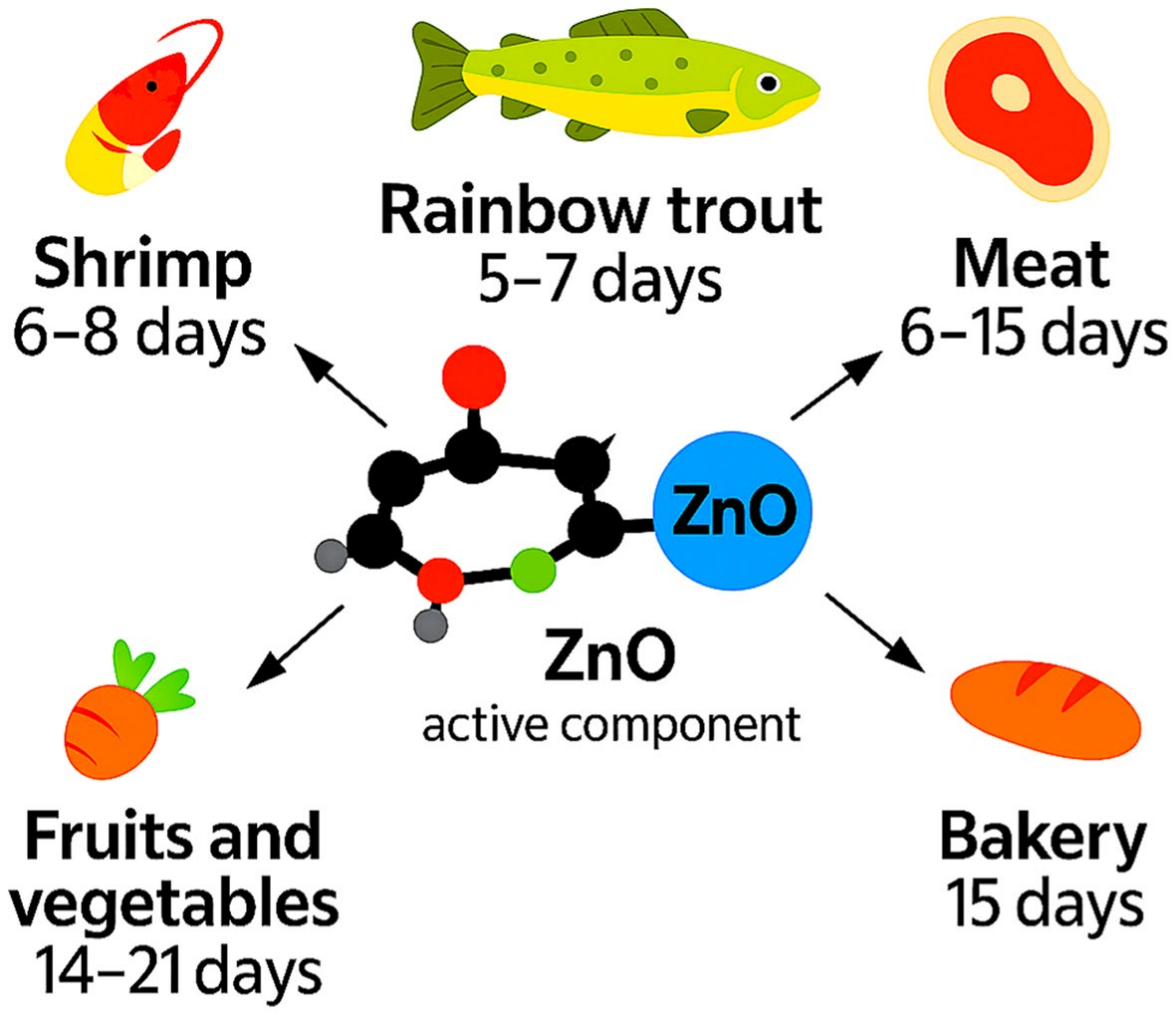
Shelf-life extension ranges for PHBHHx-ZnO active-packaging systems across major food categories.

Shelf-life extension in PHBHHx-ZnO systems arises from coordinated antimicrobial, antioxidant, and UV-shielding mechanisms that reduce microbial proliferation, suppress lipid oxidation, and maintain color and nutrient integrity during chilled storage (11, 16, 33, 56, 58, 59) (Kumar et al., 2019; Borah et al., 2025; Rahmanifarah et al., 2025). Figure 12 captures the shelf-life extension ranges associated with ZnO-enabled active-packaging systems across major food categories.

### 5.5 Relevance for high-risk food categories

PHBHHx-ZnO systems show strong potential for fish, meat, dairy, and bakery products, where microbial growth and oxidative deterioration drive rapid quality loss (4, 24). Studies report lower microbial loads, slower oxidation, and improved sensory attributes during chilled storage when PHBHHx-ZnO films are used (17, 25). PHBHHx matrices provide flexibility, biodegradability, and stable nanoparticle dispersion, which support consistent functional performance across high-risk food categories (18, 19). UV-blocking behavior from ZnO further supports the storage of light-sensitive foods such as dairy products and fresh produce (23, 38).

Comparative studies across PHBV, PLA, cellulose, and starch systems confirm that ZnO delivers similar antimicrobial, antioxidant, and UV-protective benefits across diverse biopolymer matrices (6, 27, 28, 33, 46). Smart-packaging reviews indicate that PHBHHx-ZnO is well suited to applications requiring combined antimicrobial, antioxidant, and UV-protective functions (4, 35). Biopolymer overviews confirm strong relevance for seafood, dairy, bakery, and fresh-produce storage, where active-packaging interventions extend shelf life without persistent residues (17, 24). PHBHHx-ZnO systems deliver consistent shelf-life gains across high-risk foods through antimicrobial, antioxidant, and UV-shielding mechanisms. Table 3 compares deterioration pathways, baseline shelf-life limits, and the extensions achieved with PHBHHx-ZnO packaging across major food categories.

**Table 3:** Shelf-life behavior of food categories under PHBHHx-ZnO packaging.

Food category and pathway	Baseline shelf life (limiting factors)	Extended shelf life (functional gains)	References
Fish (microbial growth, lipid oxidation, UV sensitivity)	6–8 days; rapid microbial rise; early rancidity; color loss	12–16 days; lower microbial load; slower oxidation; stable odor and color	(11, 12, 24, 25)
Meat (microbial spoilage, pigment oxidation)	3–5 days; discoloration; surface microbial increase	6–10 days; reduced spoilage organisms; slower pigment loss	(16, 25)
Dairy (photo-oxidation, microbial instability)	7–10 days; light-driven oxidation; off-flavor formation	14–18 days; UV protection; slower oxidative change	(4, 38)
Bakery (staling, fungal growth)	5–7 days; moisture migration; mold onset	10–14 days; delayed staling; lower fungal load	(17, 27)
Fresh produce (browning, pigment loss, dehydration)	4–8 days; color fading; moisture loss	8–16 days; improved firmness; reduced pigment loss; slower respiration	(4, 25)

## 6 Safety, regulatory considerations, and environmental relevance of PHBHHx-ZnO systems

### 6.1 Safety in food-contact applications

ZnO nanoparticles represent a well-studied antimicrobial and UV-active additive in food-contact matrices, with safety outcomes shaped by dose, dispersion, and migration behavior (6, 28, 33, 46). Biopolymer matrices with moderate crystallinity show lower nanoparticle mobility than amorphous systems, which aligns with migration trends reported for PLA, PHBV, and cellulose (6, 28). PHBHHx films prepared with defined ZnO types show tight morphology and uniform dispersion, which supports stable interfaces and helps limit release (22, 26). PHBHHx-ZnO films with narrow particle-size distributions show more stable interfaces and lower release values than systems containing broad distributions (22, 26).

Application-oriented trials in seafood and beverages show antimicrobial efficacy at modest ZnO loadings, with no detectable nanoparticle migration above regulatory thresholds (11, 12). These studies confirm that ZnO remains immobilized in the polymer matrix during chilled storage, with antimicrobial benefits achieved without exceeding migration limits (11, 12). Formulation discipline, particle size, surface chemistry, and controlled distribution remain central to safe performance (33, 46). PHBHHx matrices provide additional safety advantages because flexibility and moderate crystallinity support uniform nanoparticle immobilization during storage (18, 19).

Comparative active-compound systems show similar migration constraints. PHBV films containing ferulic or p-coumaric acid remain below regulatory limits, with higher release in ethanol-rich simulants and limited release in aqueous media (49). PHBV-tannin films show negligible migration during forced aqueous extraction, with tannin retention close to nominal values (43). These systems illustrate how polymer crystallinity, additive chemistry, and dispersion govern migration across PHA matrices. Recent reviews note that surface-engineered ZnO, doped structures, and green-synthesized nano-ZnO may further reduce migration and strengthen safety profiles in biopolymer matrices (5, 29, 40). PHBHHx offers strong ZnO immobilization due to its flexibility, moderate crystallinity, and uniform dispersion. Table 4 maps migration behavior, influencing factors, and safety outcomes for ZnO in PHBHHx and related biopolymer matrices.

**Table 4:** Migration behavior and safety outcomes for ZnO in biopolymer matrices.

Matrix and migration pathway	Migration trend and influencing factors	Safety outcome and regulatory relevance	References
PLA (surface-active migration, acid-sensitive foods)	Moderate migration at 3–5%; influenced by acidity, fat content and particle agglomeration	Occasional threshold exceedance; requires controlled placement or coated formats	(12, 38)
PHBV (crystalline confinement, limited mobility)	Low migration at 2–4%; crystallinity restricts Zn^2+^ diffusion	Rare exceedance; stable in multilayer structures	(31, 32)
PHBHHx (functional-layer confinement, uniform dispersion)	Very low migration at 1–3%; dispersion and crystallinity immobilize ZnO	No exceedance reported; suitable for chilled foods and multilayer builds	(19, 22)

### 6.2 Regulatory frameworks for active and intelligent packaging

Active and intelligent packaging operates within regulated frameworks that require migration studies, toxicological assessments, and validated functional stability (4, 25). Regulatory assessments emphasize the need for matrix-specific migration data because nanoparticle mobility varies strongly with polymer crystallinity and food polarity (4, 25). Recent reviews of chemical-safety issues in emerging packaging highlight compliance pathways and analytical challenges, including detection limits for nanoparticle-related species and matrix-specific extraction conditions (50).

Analytical reviews also highlight the difficulty of distinguishing ionic Zn from nanoparticulate ZnO during migration testing, which complicates regulatory interpretation (50). Practical compliance strategy for PHBHHx-ZnO systems centers on modest particle loadings, verified dispersion, and functional-layer placement when reduced direct food contact is required (22, 23). Functional-layer placement within multilayer films provides a recognized compliance route for nano-enabled packaging (22, 23). Regulatory guidance for fish, meat, and dairy packaging increasingly recognizes the role of active layers that remain physically separated from direct food contact while still delivering antimicrobial and UV-protective functions (4, 25).

Smart-packaging regulatory literature also identifies PHBHHx-ZnO as compatible with future sensing and indicator technologies when functional layers limit migration exposure (5, 35). PHBHHx-ZnO systems maintain environmental compatibility across compost, soil, and marine settings due to uniform ZnO immobilization and efficient enzymatic degradation. Table 5 outlines environmental behavior, degradation pathways, and end-of-life outcomes for PHBHHx-ZnO and related matrices.

**Table 5:** Environmental fate and biodegradation behavior of PHBHHx-ZnO and related matrices.

System and degradation pathway	Behavior and influencing factors	Outcome and environmental relevance	References
PLA (hydrolysis-driven degradation)	Slow breakdown in soil; faster under industrial composting; ZnO migration influenced by moisture	Moderate environmental compatibility; requires controlled composting	(7, 8)
PHBV (enzyme-mediated degradation)	Steady degradation in compost and soil; crystallinity slows early stages; ZnO remains confined	High compatibility; predictable breakdown under aerobic conditions	(18, 32)
PHBHHx (esterase-driven degradation with flexible chains)	Rapid microbial access; uniform ZnO dispersion supports immobilization during early stages	Strong environmental relevance; stable degradation in compost, soil, and marine settings with no evidence of nanoparticle release above regulatory thresholds	(22, 31)

### 6.3 Environmental relevance and end-of-life behavior

PHBHHx materials show robust biodegradation under industrial composting, with documented progression in soil and marine settings through microbial depolymerases (7, 8). PHA family biodegradation proceeds through microbial depolymerases that remain active across compost, soil, and marine environments, which positions PHBHHx within a well-documented degradation pathway (7, 8). Low ZnO fractions show no inhibition of biodegradation in comparable biopolymer matrices, and immobilization within crystalline regions during early phases reduces release risk (18, 31). ZnO particles embedded within crystalline regions show delayed release during early biodegradation, which aligns with immobilization trends observed in PHBV and PLA matrices (18, 31).

Environmental caution remains necessary because excessive nanoparticle accumulation may disrupt microbial communities, which reinforces the need for optimized ZnO fractions (27, 28). PHBHHx production now links to renewable feedstocks and volatile-fatty-acid routes from food-waste fermentation, with scale-up progress reported across PHA production platforms (15, 51). Biodegradation studies on PHBHHx reinforced with natural compounds confirm that bioactive additives do not impede compost disintegration and maintain compatibility with microbial breakdown pathways (18).

Comparative PHBV-tannin films show similar behavior, with tannins retained strongly within the polymer even under extended aqueous extraction, supporting stable behavior during early degradation (43). PHBV-phenolic-acid systems also maintain biodegradation compatibility, with active compounds not impeding compost disintegration (49). Life-cycle analyses indicate alignment between PHBHHx-ZnO and sustainable disposal pathways when renewable feedstocks, efficient processing, and compostable architectures are used (9, 52).

### 6.4 Alignment with sustainability priorities

Shelf-life extension provides a direct lever against food loss and waste, and active films that combine antimicrobial control, UV shielding, and oxidative-stability support show meaningful gains across bakery and seafood case studies under chilled storage (4, 11). Case studies in bakery and seafood systems show measurable reductions in spoilage-related waste when active films are used under chilled storage (4, 11). Sector guidance for fresh meat and fish underscores dual objectives, lowered spoilage and reduced packaging persistence, where PHBHHx-ZnO systems align through functional layers and biodegradable end-of-life behavior (17, 25).

Life-cycle assessments report favorable environmental footprints for biopolymer packaging when renewable feedstocks, efficient processing, and appropriate end-of-life routes are used (52). Broader sustainability reviews highlight rising research and industrial interest in biobased plastics as part of global strategies to reduce plastic persistence and food waste (9, 10). Emerging sustainability pathways further highlight PHBHHx-ZnO compatibility with circular-economy models, including renewable-feedstock PHA production, functional-layer placement, and smart-packaging architectures (5, 35). Overall alignment emerges through reduced food waste, lower reliance on persistent plastics, and credible end-of-life outcomes under managed conditions (7, 8). PHBHHx-ZnO systems sit at the intersection of food protection, material safety, and environmental responsibility, supporting transitions toward circular packaging models.

## 7 Comparison of PHBHHx-ZnO systems with other biodegradable nanocomposites

### 7.1 Comparison with PLA-based nanocomposites

PLA-ZnO nanocomposites present strong mechanical strength and good transparency, but brittleness and limited flexibility restrict performance in applications that require repeated handling or deformation (23, 46). PHBHHx-ZnO nanocomposites present stronger flexibility due to lower crystallinity and higher chain mobility, which supports broader use in flexible and semirigid packaging formats (18, 19).

PLA-ZnO nanocomposites offer strong UV protection and antimicrobial activity, but PHBHHx-ZnO nanocomposites present more balanced mechanical and barrier behavior because of stronger polymer-nanoparticle interactions (23, 46). PLA matrices also degrade more slowly in soil and marine settings, whereas PHBHHx matrices show faster and more consistent biodegradation due to microbial enzymatic activity (7, 8). Higher moisture sensitivity in PLA-ZnO systems further limits performance in chilled and high-humidity foods (44, 61).

Comparative PHA-based systems provide additional benchmarks for multifunctionality. PHBV films containing ferulic or p-coumaric acid deliver oxygen-barrier gains of around 10–35% and enhanced UV blocking, while PHBV-tannin films provide strong UV shielding and approximately 5–10% WVTR reductions with negligible migration during forced extraction (43, 49). These molecular-additive systems illustrate how crystallinity and polymer-additive interactions shape barrier performance and multifunctionality in biodegradable matrices, and they highlight why PHBHHx-ZnO maintains more stable nanoparticle immobilization than PLA under acidic or high-polarity conditions (50).

### 7.2 Comparison with PHBV-based nanocomposites

PHBV-ZnO nanocomposites present strong mechanical strength and good barrier behavior, but brittleness and narrow processing windows restrict industrial scalability (18, 31). PHBHHx-ZnO nanocomposites present broader processing windows due to lower crystallinity and higher thermal stability, which supports extrusion, film-casting, and fiber-spinning routes (19, 22). PHBV matrices present strong oxygen-barrier behavior, but PHBHHx-ZnO nanocomposites present more balanced performance due to improved flexibility, stronger interfacial adhesion, and more stable nanoparticle dispersion (18, 31).

PHBV-ZnO systems typically show higher haze and lower transparency than PHBHHx-ZnO systems, which restricts their suitability for visible-product packaging (23, 38). Structural studies also report greater ZnO agglomeration in PHBV at moderate loadings, whereas PHBHHx matrices disperse ZnO more uniformly because of their lower crystallinity and higher chain mobility (28). Molecular-additive systems in PHBV provide additional structural benchmarks. Tannin-based films reduce OTR by around 10–35% and enhance UV blocking through hydrogen bonding and aromatic absorption (43).

PHBV films containing ferulic or p-coumaric acid show similar barrier and UV-blocking gains, reinforcing how phenolic structures influence crystallinity and diffusion pathways (49). These tannin- and phenolic-acid systems illustrate how both molecular and nanoparticulate additives shape crystallinity, barrier behavior, and multifunctionality across PHA matrices (43, 49). Recent reviews further note that PHBHHx provides more stable ZnO immobilization than PHBV in acidic or high-polarity environments, reflecting its lower crystallinity and more flexible chain architecture (50).

### 7.3 Comparison with starch-based nanocomposites

Starch-ZnO nanocomposites present strong biodegradability and good oxygen-barrier behavior, but high moisture sensitivity restricts performance in high-humidity environments. PHBHHx-ZnO nanocomposites present lower moisture sensitivity due to hydrophobic polymer chains and stronger polymer-nanoparticle interactions (44, 61). Starch-ZnO nanocomposites present strong antimicrobial activity, but mechanical behavior remains weak due to limited chain mobility and high-water uptake (27, 33). PHBHHx-ZnO nanocomposites present stronger mechanical integrity and more stable performance during storage, which supports broader industrial adoption (27, 33). Starch matrices also show higher nanoparticle migration under humid conditions, whereas PHBHHx matrices maintain tighter interfaces and lower mobility (31, 32).

Recent reviews note similar moisture-induced migration challenges in xylan/PVA-ZnO films, reinforcing the advantage of PHBHHx in humid environments (39). Starch-based nanocomposites reinforced with phenolic acids or tannins show strong antioxidant behavior but retain high WVTR and moisture-driven migration, further highlighting the stability of PHBHHx-ZnO under chilled and high-humidity storage (44, 61). PHBV-tannin films reduce OTR by around 10–35% and enhance UV blocking through hydrogen bonding and aromatic absorption, whereas PHBHHx-ZnO achieves barrier gains primarily through nucleation-driven crystallinity increases while maintaining flexibility (31, 43).

### 7.4 Comparison with cellulose-based nanocomposites

Cellulose-ZnO nanocomposites present strong mechanical strength and good oxygen-barrier behavior, but limited flexibility restricts use in applications that require deformation or repeated handling (31, 32). PHBHHx-ZnO nanocomposites present stronger flexibility and more stable mechanical behavior due to lower crystallinity and higher chain mobility (31, 32). Cellulose-ZnO nanocomposites present strong UV protection and antimicrobial activity, but PHBHHx-ZnO nanocomposites present more balanced performance due to stronger polymer-nanoparticle interactions and more uniform dispersion (23, 28).

Cellulose matrices also show higher water uptake and faster nanoparticle release under humid conditions, whereas PHBHHx matrices maintain tighter interfaces and lower migration (31, 32). Further studies on nanocellulose-PHA composites confirm that PHBHHx offers stronger dispersion stability than other PHA-cellulose blends, reflecting its lower crystallinity and higher chain mobility (18). Starch-based nanocomposites reinforced with phenolic acids or tannins show strong antimicrobial and antioxidant behavior, but high WVTR and moisture-driven migration continue to constrain performance in humid environments (44, 61). PHBHHx-ZnO maintains lower water uptake and more stable interfaces, supporting stronger mechanical integrity and steadier storage performance under chilled and high-humidity conditions (31, 32).

### 7.5 Comparison with chitosan-based nanocomposites

Chitosan-ZnO nanocomposites present strong antimicrobial activity due to inherent cationic behavior and nanoparticle synergy, but high moisture sensitivity and limited mechanical strength restrict performance in high-humidity environments (6, 33). PHBHHx-ZnO nanocomposites present lower moisture sensitivity and stronger mechanical integrity, which supports broader application in refrigerated storage (27, 46). Chitosan matrices present strong oxygen-barrier behavior, but PHBHHx-ZnO nanocomposites present more balanced performance due to improved flexibility, stronger thermal stability and more consistent nanoparticle dispersion (27, 46). Chitosan-ZnO systems also show higher nanoparticle migration under acidic conditions, whereas PHBHHx-ZnO systems maintain lower release due to tighter morphology (31, 32).

Comparative antimicrobial analyses also show that PHBHHx-ZnO maintains activity more consistently under chilled storage than chitosan systems, which lose performance under high-humidity and acidic conditions (24, 25). Cellulose-ZnO films provide strong UV absorption and good oxygen-barrier behavior but limited extensibility for flexible formats (31, 32). PHBHHx-ZnO combines UV protection with higher extensibility and more uniform nanoparticle immobilization, reflecting its lower crystallinity, stronger interfacial adhesion, and more stable filler-matrix interfaces (23, 28).

### 7.6 Comparison with PCL-based nanocomposites

PCL-ZnO nanocomposites present strong flexibility and good processability, but slow biodegradation restricts environmental relevance (7, 8). PHBHHx-ZnO nanocomposites present faster biodegradation under soil, marine, and composting conditions due to microbial enzymatic activity (7, 8). PCL-ZnO nanocomposites present moderate mechanical strength and limited oxygen-barrier behavior, whereas PHBHHx-ZnO nanocomposites present stronger mechanical integrity and more balanced barrier performance due to improved crystallinity and stronger polymer-nanoparticle interactions (31, 32). PCL matrices also show weaker UV protection at comparable ZnO loadings, whereas PHBHHx-ZnO systems show stronger UVA and UVB absorption due to tighter dispersion (23, 38).

Advances in green-synthesized ZnO also show stronger UV-shielding compatibility with PHBHHx than with PCL (40). Chitosan-ZnO systems provide strong antimicrobial activity and good film formation, yet hydrophilicity drives rapid release in acidic simulants and reduces mechanical stability under humid conditions (6, 33). PHBHHx-ZnO shows lower moisture sensitivity and more consistent mechanical behavior during chilled storage, with reduced nanoparticle migration due to tighter morphology (31, 32). PCL-ZnO films offer flexibility and processability, but slower biodegradation and weaker barrier performance limit suitability for high-risk foods (7, 8). PHBHHx-ZnO degrades faster in compost, soil, and marine settings and provides stronger oxygen-barrier and UV-shielding performance due to improved crystallinity and dispersion (31, 38).

### 7.7 Processing windows and industrial scalability

PHBHHx-ZnO nanocomposites present broader processing latitude than many biodegradable matrices because lower crystallinity and higher thermal stability support extrusion, blown-film processing, casting and fiber-spinning routes (19, 22). PHB and PHBV systems show narrower melt windows and higher shear sensitivity, which restrict scalability and increase the risk of thermal degradation during industrial processing (8, 42). PHBHHx-ZnO structures maintain stable viscosity, stronger nucleation control, and more uniform morphology under cooling, which supports consistent film formation and fiber production (22, 26). These characteristics strengthen the industrial relevance of PHBHHx-ZnO systems for the large-scale manufacture of active packaging formats.

Recent industrial reviews highlight PHBHHx as one of the most scalable PHA matrices for active packaging due to its wide melt-processing windows and stable nanoparticle dispersion (5, 47). PHBHHx-ZnO maintains stable viscosity, broad melt-processing latitude, and strong nucleation control, supporting industrial extrusion, coatings, and fiber spinning (19, 22). In contrast, PHB and PHBV show narrower melt windows and higher shear sensitivity, which increases degradation risk during scale-up (8, 42). PLA/PHB blends processed at 453 K improve melt stability relative to neat PLA, yet still do not match the processing robustness and morphology control achieved in PHBHHx-ZnO systems (26, 53). PHBHHx-ZnO offers the broadest processing latitude and most stable morphology control

## 8 Emerging trends, challenges, and future directions

### 8.1 Emerging trends

Green ZnO routes now occupy central attention, with plant extracts, microbially mediated processes, and waste-derived precursors reported for lower energy footprints and improved regulatory acceptance (23, 30, 40). Surface-engineered ZnO particles, including ligand-stabilized, doped, and core-shell forms, present steadier dispersions, sharper nucleation, and more controlled Zn2 + release in biopolymer films (5, 29). Hybrid ZnO constructs that incorporate natural phenolics or carbon-based domains present broader antioxidant capacity while retaining food-safe performance windows (27, 33). Multilayer and coated architectures with active cores or functional faces present tailored barrier behavior, antimicrobial baselines, and clear optics when particle placement remains disciplined (22, 26). PHBHHx and related PHAs perform well in these stacks, with UV-blocking and oxygen-barrier gains recorded at modest ZnO loadings (31, 38). PHBHHx-ZnO films also show crystallinity around 53–56%, which supports stable optical and mechanical behavior in multilayer formats (22).

Smart-packaging elements such as pH-responsive color, fluorescence readouts, and spoilage indicators now progress from laboratory demonstrations toward practical use in fish, meat, and bakery sectors (4, 25, 35). Electrospun mats and fiber-based formats based on PHBV, PLA, and related biopolymers present expanded surface area and active contact coverage for contact-intensive foods, with processing stability and regulatory considerations now cataloged for food-contact deployment (19, 22, 32).

Broader packaging reviews document a shift from single-function films toward multifunctional active stacks that combine antimicrobial, antioxidant, and sensing capabilities (5, 35). These trends align with sector priorities for chilled fish, dairy, and fresh produce, where combined UV, antimicrobial, and oxidative-stability functions support extended shelf life (4, 38). Comparative results from PHBV-phenolic-acid and PHBV-tannin systems confirm that biobased molecular additives can complement ZnO nanoparticulate behavior, offering additional routes to UV absorption, antioxidant activity, and controlled migration (43, 49). Recent analyses identify PHBHHx as one of the most adaptable PHA matrices for smart-packaging integration due to its flexible processing window and stable nanoparticle immobilization (47, 48).

### 8.2 Key challenges

Agglomeration at higher ZnO fractions remains a recurrent constraint, as reduced dispersion quality lowers ductility, raises haze, and interrupts barrier gains (22, 26). Migration control and dose discipline remain central regulatory themes for active materials, with fatty or acidic foods requiring conservative loadings and validated analytical methods (25, 50). Environmental fate and microbial-community impact remain important considerations, as low ZnO fractions are tolerated in many matrices, whereas excessive accumulation may disturb microbial ecologies (27, 28). PHBHHx matrices immobilize ZnO within crystalline regions during early degradation, but excessive loadings still risk release under compost or soil conditions (18, 31).

Industrial scalability remains constrained by narrower melt windows in PHA families than in PLA, sensitivity to residence time and shear, and bottlenecks in fiber spinning at high throughput (8, 19). Cost and reproducibility remain practical challenges, as greener ZnO routes and hybrid particles introduce additional preparation steps, and consistent particle type and layer placement remain essential for factory-level repeatability (23, 35). Life-cycle and footprint verification remains necessary, as the advantages of biopolymers over conventional plastics depend on feedstocks, energy intensity, and end-of-life routing (9, 52). Analytical challenges persist in distinguishing ionic Zn from nanoparticulate ZnO during migration testing, which complicates regulatory interpretation (50).

Comparative work in PHBV-tannin and PHBV-phenolic-acid systems confirms that migration behavior varies strongly with additive polarity and matrix chemistry, reinforcing the need for matrix-specific assessments (43, 49). Emerging work highlights the need for standardized nanoparticle-sensing protocols and validated Zn-speciation analytics to support regulatory approval (4, 35).

### 8.3 Future directions

Stable incorporation and controlled release remain priority goals, with surface-engineered ZnO, plant-mediated particles, and hybrid constructs offering routes to maintain activity while respecting migration thresholds (22, 23, 27). Standardized migration and sensing protocols remain essential for nano-enabled active packaging, with harmonized methods and shelf-life metrics required for seafood and meat chains (4, 25). Life-cycle-first design now guides material choices, with LCA-tuned decisions on particle preparation, layer architecture, and disposal routes, supported by circular-economy strategies that incorporate renewable feedstocks and waste valorization (15, 52).

Wider material scope emerges through lignin nanoparticles and other bio-based fillers, which present mechanical and barrier enhancements in food-contact films (47, 48). Process-level safeguards remain essential, with disciplined cooling, short residence times, and clean die design required to preserve viscosity and crystallization profiles during scale-up (19, 42).

Sector-specific playbooks now guide chilled fish and fresh-fruit chains, where combined antimicrobial, UV, and antioxidant functions align with postharvest handling protocols for additive shelf-life gains (4, 25). Future PHBHHx-ZnO systems will likely integrate hybrid fillers, functional layers, and sensing elements within recyclable or compostable architectures, supporting transitions toward low-waste, high-performance packaging. Recent directions also suggest the integration of electrospun sensing meshes, active indicator layers, and pH-responsive colorimetric systems into PHBHHx stacks for real-time freshness monitoring (5, 35). These approaches align with broader trends toward multifunctional biopolymer platforms that combine barrier performance, antimicrobial activity, and embedded sensing. Continued advances in nano-enabled signal amplification, low-migration dye carriers, and biodegradable conductive pathways will further support the development of PHBHHx-based smart-packaging architectures capable of real-time quality tracking and end-of-life biodegradability.

## 9 Conclusion

This review examines the structural, functional, and ecological evidence that establishes PHBHHx-ZnO as a next-generation platform for sustainable active food packaging. The combined system unites mechanical reinforcement, barrier stability, antimicrobial potency, ultraviolet shielding, antioxidant capacity, and full biodegradability within one material architecture. PHBHHx-ZnO films show crystallinity around 53–56%, stable dispersion, low haze, and consistent performance across films, fibers, and multilayer structures. Comparative studies across starch, gelatine, PLA, agar, chitosan, alginate, cellulose, and PHBV demonstrate shelf-life extension in dairy, meat, fish, produce, and bakery applications, confirming the broad relevance of ZnO-based bionanocomposites. Hybrid systems that incorporate essential oils, phenolic compounds, or metallic dopants deliver stronger antimicrobial and antioxidant performance, expanding the functional scope of ZnO-reinforced films and strengthening their active preservation capacity during chilled storage. Advances in industrial biotechnology and green ZnO synthesis provide credible pathways for scalable and ecologically compatible PHBHHx-ZnO production, while life-cycle assessments align the system with circular-economy objectives. PHBHHx matrices support renewable-feedstock production routes and maintain biodegradation across compost, soil, and marine environments even at low ZnO loadings. Safe adoption will depend on harmonized regulatory frameworks, comprehensive migration studies, and continued optimization of synthesis routes to balance functional performance with toxicological control. Functional-layer placement, disciplined particle dispersion, and controlled Zn2 + release remain central to compliance. The evidence indicates that PHBHHx-ZnO functions as an active bio-packaging platform, with ZnO-driven antimicrobial, antioxidant, and UV-shielding mechanisms directly intervening in spoilage pathways and supporting measurable gains in food safety and shelf life. Moreover, the findings show that PHBHHx-ZnO can serve as a robust, adaptable, and environmentally responsible foundation for next-generation active-packaging technologies, capable of reducing food waste, lowering persistent-plastic burdens, and supporting sustainable supply-chain transitions. Future progress will depend on harmonized analytics, disciplined material design, and integrated circular-economy strategies that link food protection with environmental responsibility.
